# Supplementary material for: Bacterial profile and antimicrobial susceptibility patterns of isolates from inanimate objects used by healthcare professionals at Debre Markos Comprehensive Specialized Hospital, Northwest Ethiopia
Source: PLoS One. 2024 Nov 11;19(11):e0313474. doi: 10.1371/journal.pone.0313474 (PMC11554123; doi:10.1371/journal.pone.0313474)
Supplement: S1 Table — (DOCX) [file pone.0313474.s001.docx]

Table 9: Multidrug resistance pattern of *Pseudomonas aeruginosa* isolates from inanimate objects used by healthcare professionals, at DMCSH, Northwest Ethiopia 2023

| Name of Antimicrobials | No of classes non-susceptible | *P. aeruginosa* (n=18) | MDR N(%) |
| --- | --- | --- | --- |
| None | 0 | 1(5.6) | - |
| PIP | 1 | 3(16.7) | - |
| CAZ, GEN*, TOB*, | 2 | 1(5.6) | - |
| PIP, CIP, CAZ | 3 | 1(5.6) | 1(5.6) |
| AMK*, CAZ, PIP, CIP | 4 | 1(5.6) | 1(5.6) |
| CAZ, PIP, MEM, CIP | 4 | 1(5.6) | 1(5.6) |
| AMK, CAZ, PIP, CIP | 4 | 1(5.6) | 1(5.6) |
| CAZ, PIP, GEN*, TOB*, CIP | 4 | 2(11.1) | 2(11.1) |
| CAZ, PIP, GEN*, TOB*, CIP | 4 | 4(22.2) | 4(22.2) |
| CAZ, PIP, MEM, GEN*, TOB*, | 4 | 1(5.6) | 1(5.6) |
| AMK*, CAZ, PIP, GEN*, CIP | 4 | 1(5.6) | 1(5.6) |
| AMK*, CAZ, PIP, GEN*, TOB*, CIP | 4 | 1(5.6) | 1(5.6) |
| Total |  | 18(100) | 13(72.2) |

^Key:^ ***^:^** ^the same class of drugs,^ ^AMK: Amikacin,^ ^CIP: Ciprofloxacin, CN: Gentamicin, CAZ: Ceftazidime, MEM: Meropenem, FEP: Cefepime, PIP: piperacillin, TOB: tobramycin^
