# Supplementary material for: Bacterial profile and antimicrobial susceptibility patterns of isolates from inanimate objects used by healthcare professionals at Debre Markos Comprehensive Specialized Hospital, Northwest Ethiopia
Source: PLoS One. 2024 Nov 11;19(11):e0313474. doi: 10.1371/journal.pone.0313474 (PMC11554123; doi:10.1371/journal.pone.0313474)
Supplement: S2 Table — (DOCX) [file pone.0313474.s002.docx]

Table 10: Multidrug resistance pattern of Gram-positive isolates from inanimate objects used by healthcare professionals at DMCSH, Northwest Ethiopia 2023.

| Name of Antimicrobials | No of classes non-susceptible | *S. aureus* (n=83) | *S. epidermidis* (n=64) |
| --- | --- | --- | --- |
| None | - | 2(2.4) | 1(1.6) |
| PEN | 1 | 5(6) | 8(12.5) |
| PEN, DOX | 2 | 3(3.6) | 5(7.8) |
| PEN, AZM |  | 5(6) | 3(4.7) |
| PEN, CHL |  | 3(3.6) | 1(1.6) |
| PEN, SXT |  | 12(9.6) | 3(4.7) |
| PEN, SXT, CHL | 3 | 1(1.2) | 1(1.6) |
| PEN, SXT, AZM |  | 6(7.2) | 4(6.3) |
| PEN, FOX, SXT, DOX | 4 | 5(6) | - |
| PEN, OX, CIP, SXT |  | - | 2(3.1) |
| PEN, AZM, DOX, SXT |  | 3(3.6) | 4(6.3) |
| PEN, CIP, GEN, SXT |  | 1(1.2) | 1(1.6) |
| PEN, SXT, AZM, CHL |  | 1(1.2) | 1(1.6) |
| PEN, CIP, SXT, DOX |  | 1(1.2) | 2(3.1) |
| PEN, CIP, FOX, CHL, DOX | 5 | 1(1.2) | - |
| PEN, FOX, SXT, AZM, CIP |  | 2(2.4) | - |
| PEN, OX, SXT, AZM, DOX |  | - | 1(1.6) |
| PEN, GEN, CIP, CHL, SXT |  | 3(3.6) | 1(1.6) |
| PEN, AZM, FOX, CIP, DOX |  | 1(1.2) | - |
| PEN, CIP, AZM, DOX, CHL |  | 3(3.6) | 1(1.6) |
| PEN, OX, SXT, CLN, DOX |  | - | 2(3.1) |
| PEN, CIP, OX, GEN, DOX |  | - | 3(4.7) |
| PEN, DOX, CHL, FOX, GEN |  | 3(3.6) | - |
| PEN, FOX, SXT, AZM, DOX |  | 7(8.4) | - |
| PEN, FOX, GEN, AZM, CHL |  | 1(1.2) | - |
| PEN, FOX, SXT, AZM, CHL |  | 1(1.2) | - |
| PEN, AZM, OX, CIP, DOX, SXT | 6 | - | 1(1.6) |
| PEN, OX, CIP, SXT, DOX, CLN |  | - | 1(1.6) |
| PEN, OX, SXT, AZM, GEN, CIP |  | - | 5(7.8) |
| PEN, CIP, SXT, CLN, AZM, GEN |  | 1(1.2) | 1(1.6) |
| PEN, FOX, CIP, SXT, DOX, CLN |  | 1(1.2) | 2(3.1) |
| PEN, FOX, CHL, SXT, DOX CIP |  | 1(1.2) | - |
| PEN OX, CIP, SXT, DOX, AZM |  | - | 1(1.6) |
| PEN, OX, CHL, SXT, DOX, AZM |  | - | 4(6.3) |
| PEN, CIP, SXT, AZM, CHL, DOX |  | 1(1.2) | 2(3.1) |
| PEN, GEN, SXT, CLN, AZM, FOX |  | 1(1.2) | - |
| PEN, GEN, SXT, CLN, AZM, CHL |  | 2(2.4) | 2(3.1) |
| PEN, FOX, SXT, CLN, AZM, DOX |  | 1(1.2) | - |
| PEN, FOX, CIP, SXT, AZM, DOX |  | 3(3.6) | - |
| PEN, FOX, CIP, SXT, AZM, CHL |  | 2(2.4) | - |
| PEN, OX, SXT, AZM, CHL, DOX |  | - | 1(1.6) |
| Total N (%) |  | 83(100) | 64 (100) |

**^Key^**^: CHL: Chloramphenicol, PEN: Penicillin, CIP: Ciprofloxacin, CN: Gentamicin, FOX: Cefoxitin, SXT: Sulfametexazole/trimetoprim, CLN: Clindamycin, DOX: Doxycycline, AZM: Azithromycin, OX: Oxacillin^
