# Supplementary material for: Bacterial profile and antimicrobial susceptibility patterns of isolates from inanimate objects used by healthcare professionals at Debre Markos Comprehensive Specialized Hospital, Northwest Ethiopia
Source: PLoS One. 2024 Nov 11;19(11):e0313474. doi: 10.1371/journal.pone.0313474 (PMC11554123; doi:10.1371/journal.pone.0313474)
Supplement: Attachment — Submitted filename: Commented PONE-D-24-37007_reviewer (1).pdf [file pone.0313474.s004.pdf]

PLOS ONE

Bacterial profile and antimicrobial susceptibility patterns of isolates from inanimate objects used by healthcare professionals and associated factors at Debre Markos Comprehensive Specialized Hospital, Northwest Ethiopia

--Manuscript Draft--

**Manuscript Number:**	PONE-D-24-37007
**Article Type:**	Research Article
**Full Title:**	Bacterial profile and antimicrobial susceptibility patterns of isolates from inanimate objects used by healthcare professionals and associated factors at Debre Markos Comprehensive Specialized Hospital, Northwest Ethiopia
**Short Title:**	Bacterial profile and antimicrobial susceptibility pattern from inanimate objects in a hospital
**Corresponding Author:**	Tebelay Dilnessa, MSc<br>Debre Markos University<br>Debre Markos, ETHIOPIA
**Keywords:**	Antimicrobial susceptibility test, Contamination, Inanimate objects, Healthcare-associated infection
**Abstract:**	Background: Healthcare-associated infections occur in patients under medical care, which is a major public health issue in hospitals worldwide. The prevalence is two to three folds higher in developing countries compared to developed countries. Inanimate objects used by healthcare professionals such as gowns, mobile phones, and stethoscopes are highly prone to bacterial contamination from the healthcare setting.<br>Objective: To determine the bacterial profile, associated factors, and antimicrobial susceptibility patterns from inanimate objects used by healthcare professionals at Debre Markos Comprehensive Specialized Hospital (DMCSH), Northwest Ethiopia.<br>Methods: A cross-sectional study was conducted at Debre Markos Comprehensive Specialized Hospital from inanimate objects from April 1, 2023 to June 30, 2023 using simple random sampling technique with lottery method. Socio-demographic data was collected using a structured questionnaire. A swab sample from healthcare professionals' gowns, mobile phones, and stethoscopes were collected and inoculated onto blood agar, chocolate and MacConkey agar. Isolates were identified and characterized by colony morphology, Gram staining and biochemical tests. Antimicrobial susceptibility testing was performed in all isolates by the disk diffusion technique on Muller Hinton agar according to CLSI, 2022 guidelines. Data was entered into EPI-Data and analyzed using SPSS version 25. Logistic regression model was used. Variables with p-value ≤ 0.25 in bivariable logistic regression was fitted to multivariate logistic regression analysis. A p-value of ≤ 0.05 with 95%CI was considered statistically significant.<br>Result: A total of 376 healthcare professionals' inanimate objects were included. Overall, 60.1% (226/376) inanimate objects were contaminated. The proportion of bacterial contamination was (65.1%; 82/126), (60.3%; 76/126) and (54.8%, 68/124) for mobile phones, gowns and stethoscopes, respectively. Staphylococcus aureus was the most frequent isolate accounting (22.1%, 83/376) followed by Staphylococcus epidermidis (17.0%, 64/376), Escherichia coli (8.8%, 33/376) and Pseudomonasaeruginosa (4.9%; 18/376). Working in gynecology/ obstetrics wards (AOR: 8.69; 95%CI: 1.09-69.41, P=0.041), did not disinfect mobile phones (AOR: 2.69; 95%CI: 1.15-6.25; P=0.021) and stethoscopes regularly (AOR: 3.06, 95%CI: 1.23-7.59; P=0.016), carry mobile phones with patient care materials (AOR: 2.72; 95%CI: 1.18-6.29, P=0.019) and not taken infection prevention training (AOR:3.91; 95%CI:1.71-8.93; P=0.001) were significantly associated with bacterial contamination. Most Gram-negative bacteria were resistant to ampicillin, trimethoprim-sulfamethoxazole and amoxacilline-clavunic acid, while Gram-positive isolates showed high level of resistant to penicillin. On the other hand, meropenem, for Gram-negative and clindamycin for Gram-positive bacteria showed lower level of resistance. Multidrug resistance among Gram-positive and Gram-negative bacteria were (65.3%; 96/147) and (75.3%; 64/85), respectively with over all MDR (69%, 160/232).<br>Conclusion: Inanimate objects commonly used by healthcare professionals are important sources of bacterial contamination. Most Gram-negative bacteria were

Powered by Editorial Manager® and ProduXion Manager® from Aries Systems Corporation

	resistant to ampicillin while Gram-positive isolates showed high level of resistant to penicillin. All healthcare professionals should regularly disinfect their inanimate objects to prevent bacterial colonization and potential spread of infection.
Order of Authors:	Senedu Kindie, MSc
	Getachew Mengistu, MSc
	Mulatu Kassahun, Mph
	Abebaw Admasu, MSc
	Tebelay Dilnessa, MSc
Additional Information:	
**Question**	**Response**
**Financial Disclosure**<br><br>Enter a financial disclosure statement that describes the sources of funding for the work included in this submission. Review the submission guidelines for detailed requirements. View published research articles from *PLOS ONE* for specific examples.<br><br>This statement is required for submission and **will appear in the published article** if the submission is accepted. Please make sure it is accurate.<br><br>**Funded studies**<br>Enter a statement with the following details:<br>• Initials of the authors who received each award<br>• Grant numbers awarded to each author<br>• The full name of each funder<br>• URL of each funder website<br>• Did the sponsors or funders play any role in the study design, data collection and analysis, decision to publish, or preparation of the manuscript?<br><br>Did you receive funding for this work?	The author(s) received no specific funding for this work.
**Competing Interests**<br><br>Use the instructions below to enter a competing interest statement for this submission. On behalf of all authors, disclose any competing interests that could be perceived to bias this work—acknowledging all financial support	The authors have declared that no competing interests exist.

and any other relevant financial or non-financial competing interests.<br><br>This statement is required for submission and **will appear in the published article** if the submission is accepted. Please make sure it is accurate and that any funding sources listed in your Funding Information later in the submission form are also declared in your Financial Disclosure statement.<br><br>View published research articles from *PLOS ONE* for specific examples.<br><br>**NO authors have competing interests**<br><br>Enter: *The authors have declared that no competing interests exist.*<br><br>**Authors with competing interests**<br><br>Enter competing interest details beginning with this statement:<br><br>*I have read the journal's policy and the authors of this manuscript have the following competing interests: [insert competing interests here]*<br><br>* typeset	
**Ethics Statement**<br><br>Enter an ethics statement for this submission. This statement is required if the study involved:<br><br>• Human participants<br>• Human specimens or tissue<br>• Vertebrate animals or cephalopods<br>• Vertebrate embryos or tissues<br>• Field research<br><br>Write "N/A" if the submission does not require an ethics statement.<br><br>General guidance is provided below.	N/A

Powered by Editorial Manager® and ProduXion Manager® from Aries Systems Corporation

Consult the submission guidelines for detailed instructions. **Make sure that all information entered here is included in the Methods section of the manuscript.**<br><br>**Format for specific study types**<br><br>**Human Subject Research (involving human participants and/or tissue)**<br>• Give the name of the institutional review board or ethics committee that approved the study<br>• Include the approval number and/or a statement indicating approval of this research<br>• Indicate the form of consent obtained (written/oral) or the reason that consent was not obtained (e.g. the data were analyzed anonymously)<br><br>**Animal Research (involving vertebrate animals, embryos or tissues)**<br>• Provide the name of the Institutional Animal Care and Use Committee (IACUC) or other relevant ethics board that reviewed the study protocol, and indicate whether they approved this research or granted a formal waiver of ethical approval<br>• Include an approval number if one was obtained<br>• If the study involved *non-human primates*, add *additional details* about animal welfare and steps taken to ameliorate suffering<br>• If anesthesia, euthanasia, or any kind of animal sacrifice is part of the study, include briefly which substances and/or methods were applied<br><br>**Field Research**<br><br>Include the following details if this study involves the collection of plant, animal, or other materials from a natural setting:<br>• Field permit number<br>• Name of the institution or relevant body that granted permission	
**Data Availability**<br><br>Authors are required to make all data underlying the findings described fully available, without restriction, and from the	Yes - all data are fully available without restriction

time of publication. PLOS allows rare exceptions to address legal and ethical concerns. See the PLOS Data Policy and FAQ for detailed information.<br><br>A Data Availability Statement describing where the data can be found is required at submission. Your answers to this question constitute the Data Availability Statement and **will be published in the article**, if accepted.<br><br>**Important:** Stating 'data available on request from the author' is not sufficient. If your data are only available upon request, select 'No' for the first question and explain your exceptional situation in the text box.<br><br>Do the authors confirm that all data underlying the findings described in their manuscript are fully available without restriction?	
**Describe where the data may be found in full sentences. If you are copying our sample text, replace any instances of XXX with the appropriate details.**<br><br>• If the data are **held or will be held in a public repository**, include URLs, accession numbers or DOIs. If this information will only be available after acceptance, indicate this by ticking the box below. For example: *All XXX files are available from the XXX database (accession number(s) XXX, XXX.*).<br>• If the data are all contained **within the manuscript and/or Supporting Information files**, enter the following: *All relevant data are within the manuscript and its Supporting Information files.*<br>• If neither of these applies but you are able to provide **details of access elsewhere**, with or without limitations, please do so. For example:<br><br>*Data cannot be shared publicly because of [XXX]. Data are available from the XXX Institutional Data Access / Ethics Committee (contact via XXX) for*	All relevant data are within the manuscript and its Supporting Information files.

researchers who meet the criteria for access to confidential data.<br><br>*The data underlying the results presented in the study are available from (include the name of the third party and contact information or URL).*<br>• This text is appropriate if the data are owned by a third party and authors do not have permission to share the data.<br><br>* typeset	
Additional data availability information:	

Powered by Editorial Manager® and ProduXion Manager® from Aries Systems Corporation

Manuscript Click here to access/download;Manuscript;Manuscript.docx

Bacterial profile and antimicrobial susceptibility patterns of isolates from inanimate objects used by healthcare professionals and associated factors at Debre Markos Comprehensive Specialized Hospital, Northwest Ethiopia

Senedu Kindie [1, 2], Getachew Mengistu[1], Mulatu Kassahun [3], Abebaw Admasu [1], Tebelay Dilnessa [1]*

[1]Department of Medical Laboratory Sciences, College of Medicine and Health Sciences, Debre Markos University, Debre Markos, Ethiopia, P. O. Box 269

[2]Leul Alemayehu Health Center, Debre Tabor, Ethiopia

[3]Students' Clinic Burie Campus, Debre Markos University, Burie, Ethiopia

*Corresponding author: tebelay_dilnessa@dmu.edu.et (TD)

Abstract

Background: Healthcare-associated infections occur in patients under medical care, which is a major public health issue in hospitals worldwide. The prevalence is two to three folds higher in developing countries compared to developed countries. Inanimate objects used by healthcare professionals such as gowns, mobile phones, and stethoscopes are highly prone to bacterial contamination from the healthcare setting.

Objective: To determine the bacterial profile, associated factors, and antimicrobial susceptibility patterns from inanimate objects used by healthcare professionals at Debre Markos Comprehensive Specialized Hospital (DMCSH), Northwest Ethiopia.

Methods: A cross-sectional study was conducted at Debre Markos Comprehensive Specialized Hospital from inanimate objects from April 10, 2023 to June 30, 2023 using simple random sampling technique with lottery method. Socio-demographic data was collected using a structured questionnaire. A swab sample from healthcare professionals' gowns, mobile phones, and stethoscopes were collected and inoculated onto blood agar, chocolate and MacConkey agar. Isolates were identified and characterized by colony morphology, Gram staining and biochemical tests. Antimicrobial susceptibility testing was performed in all isolates by the disk diffusion technique on Muller Hinton agar according to CLSI, 2022 guidelines. Data was entered into EPI-Data and analyzed using SPSS version 25. Logistic regression model was used. Variables with p-value ≤ 0.25 in bivariable logistic regression was fitted to multivariate logistic regression analysis. A p-value of ≤ 0.05 with 95%CI was considered statistically significant.

Result: A total of 376 healthcare professionals' inanimate objects were included. Overall, 60.1% (226/376) inanimate objects were contaminated. The proportion of bacterial contamination was (65.1%; 82/126), (60.3%; 76/126) and (54.8%, 68/124) for mobile phones, gowns and stethoscopes, respectively. *Staphylococcus aureus* was the most frequent isolate accounting (22.1%, 83/376) followed by *Staphylococcus epidermidis* (17.0%, 64/376), *Escherichia coli* (8.8%, 33/376) and *Pseudomonas aeruginosa* (4.9%; 18/376). Working in gynecology/ obstetrics wards (AOR: 8.69; 95%CI: 1.09-69.41, *P*=0.041), did not disinfect mobile phones (AOR: 2.69; 95%CI: 1.15-6.25; *P*=0.021) and stethoscopes regularly (AOR: 3.06, 95%CI: 1.23-7.59; *P*=0.016), carry mobile phones with patient care materials (AOR: 2.72; 95%CI: 1.18-6.29, *P*=0.019) and not taken infection prevention training (AOR:3.91; 95%CI:1.71-8.93; *P*=0.001) were significantly associated with bacterial contamination. Most Gram-negative bacteria were resistant to ampicillin, trimethoprim-sulfamethoxazole and amoxacilline-clavunic

acid, while Gram-positive isolates showed high level of resistant to penicillin. On the other hand, meropenem, for Gram-negative and clindamycin for Gram-positive bacteria showed lower level of resistance. Multidrug resistance among Gram-positive and Gram-negative bacteria were (65.3%; 96/147) and (75.3%; 64/85), respectively with over all MDR (69%, 160/232).

Conclusion: Inanimate objects commonly used by healthcare professionals are important sources of bacterial contamination. Most Gram-negative bacteria were resistant to ampicillin while Gram-positive isolates showed high level of resistant to penicillin. All healthcare professionals should regularly disinfect their inanimate objects to prevent bacterial colonization and potential spread of infection.

Keywords: Antimicrobial susceptibility test, Contamination, Inanimate objects, Healthcare-associated infection

Introduction

Nosocomial infections are healthcare-associated infections (HAIs), that occur in patients during the process of acquiring healthcare services in a hospital or other healthcare facility, which was not present at the time of admission [1, 2]. Infection is frequently considered as HAI if it appears at or after 48 hours of admission and within 48-72 hours after discharge [3]. Healthcare-associated infections may also be acquired by healthcare professionals during healthcare delivery [2]. The spread of HIAs is largely due to healthcare professionals' (HCPs) contaminated hands, their inanimate objects and their contact with patients, inadequate equipment sterilization, and the rise of bacterial strains that are resistant to treatment [4]. Healthcare-associated infections to HCPs acquires during their day-to-day hospital activities such as specimen collection, processing and discarding, handling and discarding of medical equipment, and contaminated objects as well as during direct contact with the patient at the time of examination [5].

Healthcare-associated infections are more concerning in the twenty-first century for a number of reasons. Among these are hospital housing a high number of sick patients, many of whom have compromised immune systems; an increase in outpatient care; numerous medical procedures that bypass the body's natural defenses; medical staff that moves between patients allowing pathogens to spread; poor hygiene guidelines for clothes, tools, cleaning, sterilizing, and other preventive actions; and the regular use of antimicrobial agents in hospitals, which exerts selection pressure towards the emergence of resistant strains of microorganisms [6].

Healthcare-associated infections are a major health concern for millions of people globally. Hospital settings and contaminated equipment are well-known sources of illness. Healthcare professionals may act as a mobile surface for transmission due to their contaminated inanimate objects [7]. Inanimate objects that are frequently touched by hands can act as reservoirs for infections that transfer to HCP hands and ultimately to patients. These inanimate objects used by healthcare professionals pick up harmful germs and disseminate the infection to others [7, 8]. Of these inanimate objects, mobile phones, gowns and stethoscopes are highly prone to bacterial contamination from the healthcare setting and are considered potential sources for these infections. *S. aureus*, *E. coli*, *Klebsiella pneumonia*, *Proteus* species, coagulase-negative staphylococcus species, *Acinetobacter* species and *P. aeruginosa* are the most frequent bacterial isolates from HCPs mobile phones, gowns and stethoscopes [9, 10].

Currently, mobile phones have become essential accessories for HCPs and social life [11, 12]. Healthcare professionals frequently utilize mobile phones in the hospital setting for internet browsing, infusion dose calculations, and electrolyte corrections in addition to communication [13, 14]. Despite all of the potential advantages, mobile phones are known to cause illnesses linked to HAI and play a significant role in becoming potential bacterial reservoirs [15, 16]. Mobile phones have the potential to spread nosocomial infections to other locations, like workers' home [17].

Healthcare professionals' gowns become contaminated with microorganisms through regular use, which may aggravate HAIs more likely [18]. Gowns are critical elements of personal protective equipment (PPE) since they are the second-most-used piece of PPE, following gloves [19, 20]. Gowns that serve as transmission vehicles have the potential to transmit pathogens from one patient to another [21, 22].

Similarly, the stethoscope can act as an important spreader of bacterial infection among HCPs and patients [23]. When the unclean parts of the stethoscope come into direct contact with the patient's skin and the physician's hand, commonly become colonized with pathogenic isolates and subsequently be transferred to other patients if the stethoscope is not disinfected [24, 25].

Healthcare-associated infections are more aggravated once a considerable number of microorganisms are resistant to conventional antimicrobials as well as to new drugs. There is a high risk of transmission of multidrug antibiotic-resistant microorganisms in hospital settings [26, 27]. In Ethiopia, HAIs among patients increased by more than twofold between 2009 and 2018 from 5.7% to 19.41%. This indicates that there is a significant concern for patients as well as HCPs, as HAIs may raise occupational risk among HCPs [28, 29]. Despite continuing efforts of hospital infection control, HAIs are still a major public health problem globally. There is no report about the role of gowns, mobile phones, and stethoscopes in the transmisions of healthcare associated infection in the study area. Therefore, this study aimed to assess bacterial profile, associated factors, and antimicrobial susceptibility patterns from inanimate objects used by healthcare professionals at Debre Markos Comprehensive Specialized Hospital, Northwest Ethiopia.

Materials and methods

Study area and setting

The study was conducted at Debre Markos Comprehensive Specialized Hospital (DMCSH), which is found in Debre Markos town, the capital of East Gojjam zone, located 302 km Northwest of Addis Ababa, the capital city of Ethiopia, and 264 km Southeast of Bahir Dar, the capital of Amhara National Regional State. It is one of the oldest public hospitals in the country which was established in 1957. It provides health services for approximately 255,248 patients per year from a catchment population of about 5 million people. The hospital consists of an outpatient department (OPDs), emergency ward, gynecology/obstetrics, maternity, medical, surgical, orthopedics, pediatric wards, operating room, intensive care unit (ICU), laboratory and pharmacy unites. It has more than 782 staffs, including both supportive and healthcare professionals. It gives service to East Gojjam, West Gojjam, Awi zone, and some parts of the Oromia region [30].

Study design and period

A hospital-based cross-sectional study was conducted from April 10, 2023 to June 30, 2023.

Source and study population

All inanimate objects used by healthcare professionals were the source populations. Similarly, their gowns, stethoscopes and mobile phones used by these healthcare professionals were the study populations during the study period.

Dependent variable

Bacterial profiles of inanimate objects

Independent variables

Socio-demographic characteristics includes such as age, sex, year of service, field of specialization, level of education, working wards, hygiene-related practice, hand washing, disinfecting of mobile phone, use of mobile phone at bed side to medical information, answering phone calls while attending patients, carry mobile phone with patient care materials, disinfecting of stethoscope, share stethoscope, use of laundry gown, infection prevention training and infection prevention manual.

Sample size determination and sampling techniques

The sample size was calculated by using single population proportion formula based on the assumption of a 95% confidence interval ($Z\ \alpha/2 = 1.96$), 5% margin of error, and prevalence of health care worker's fomites 57.6% from previous study conducted at Felege Hiwot Referral Hospital [31].

$$n = (Z\ \alpha/2)^2 \frac{P(1-P)}{d^2}$$

Where, n=the minimum sample size.

Z= the z-value at 95 % confidence interval = 1.96;

P=the prevalence of bacteria from gowns, mobile phones and stethoscopes of HCP's=57.6%.

d=margin of sampling error taken as 5% $(1.96)^2 x \frac{0.576(1-.576)}{(0.05)2} = 376$

Therefore, a total of 376 inanimate objects were included. This sample size was proportionally allocated to different healthcare professionals and working wards according to their population size. Study participants were selected using simple random sampling technique after proportional allocation in each stratum of the occupational group. Gowns and mobile phones were collected from medical doctors, intern students, nurses, midwives, pharmacy professionals, medical laboratory professionals, radiologists, physiotherapists, ophthalmologists, dentists and dermatologists. Stethoscope were collected from health professionals who have stethoscopes by using a census until the required sample size is fulfilled because the allocated sample is larger than the physicians in the hospital (**Fig 1**).

Figure 1: Sampling technique from healthcare professionals' inanimate objects used by healthcare professionals at DMCSH, Northwest Ethiopia, 2023

Eligibility criteria

Gowns, and mobile phones of all HCPs and stethoscopes of HCPs who have stethoscope at DMCSH were included in the study. Gowns, mobile phones, and stethoscopes from HCPs on maternal and annual leave during the study period were excluded.

Operational definitions

Inanimate objects: are gown, mobile phones and stethoscopes used by healthcare professionals at DMCSH.

Hand washing: refers to cleaning hands using alcohol- or hand sanitizers or washing hands with soap and water.

Healthcare professionals: includes medical doctors, anesthetists, nurses, midwives, medical intern students, radiologists, physiotherapists, psychiatrists, dermatologists, dentists, ophthalmologists, laboratory and pharmacy professionals who work at DMCSH.

Multidrug-resistant (MDR): When a bacterial isolate is resistant to at least one drug in three or more classes of antimicrobial agents [32].

Data collection

Socio-demographic data collection

Socio-demographic and other data related to healthcare professional's gowns, stethoscopes, and mobile phones were collected by self-administered structured questionnaires.A total of 376 inanimate objects wrere enrold from April 10, 2023 to June 30, 2023. Of these gowns, mobile phones and stethoscopes 126,126 and 124 were collected, respectivly.

Specimen collection and transportation

The data collector cleaned their hands using alcohol-based hand sanitizer before taking swab samples from HCP's inanimate objects. Powder-free disposable gloves were worn per sample during the sample collection process to prevent cross-contamination. The specimen was collected by using a sterile cotton swab moistened with saline from the gowns and mobile phones of all healthcare professionals, and stethoscope swabs were collected from healthcare professionals who have stethoscopes by using a census until the required sample size was fulfilled. Specifically, a swab from the cuff of the dominant hand and pocket mouth of the gown were collected. The entire surface of the diaphragm and earpieces of each stethoscope was swabbed. From mobile phones, swab was performed on fully stretched of the screen and backside of where the most frequent areas of contact with hands. Following collection, all swab specimens were inserted into a separate test tube and labeled with sample number, sample type, time, and date of sample collection. After collection, all swab samples were transported to DMCSH Microbiology laboratory and processed within 1hour of collection.

Isolation and identification of bacteria

After the sample was aseptically collected, the moistened swab sample was inoculated onto blood agar, chocolate and MacConkey agar. All inoculated media was incubated at 35 -37^{0}C for 18 to 24 hrs. Additionally, inoculated chocolate agar media was incubated in 5% CO_2 enriched environment. Primary isolation of bacteria was made based on their colony characteristics, hemolytic pattern, Gram reaction and microscopic features. Then, further identification of bacterial organisms were analyzed with biochemical tests [33].

Biochemical tests were performed on colonies from pure cultures and inoculated onto different biochemical test media and incubated aerobically at 37ºC for 18-24 hours for identification of the isolates. The following biochemical test was tested for Gram-negative bacteria: oxidase, indole, triple sugar iron agar, citrate, urea, lysine decarboxylase (LDC) agar, motility test, and mannitol test. For Gram-positive bacteria, a catalase test was done to differentiate staphylococcal from streptococcal isolates. A slide coagulase test was done to differentiate *S. aureus* from coagulase-negative staphylococci (CoNS). Also manitol salt agar, and novobiocin was done [20, 34].

Antimicrobial susceptibility testing

The antimicrobial susceptibility testing were performed for all issolates by Kirby-Bauer disk diffusion technique on Muller-Hinton agar following the Clinical and Laboratory Standard Institute (CLSI) guideline. A loop full of bacteria (2-3 identical colonies) was taken by sterile wire loop from a pure culture colony and transferred to a test tube containing 5ml of normal saline, and the mixture was gently mixed to create a uniform suspension. The 0.5 McFarland standard was used to standardize the turbidity of the bacterial suspension [35].

Sterile cotton swabs were immersed into the suspension, remove excess fluid by gentle rotation of the swab to the inner surface of the tube, and inoculated the bacterial suspension over the entire surface of Mueller Hinton agar. The antimicrobial discs were placed and incubated at 37 ºC for 18-24 hours. After the incubation period, the diameter zone of inhibition was measured and interpreted based on CLSI 2022 guidelines. The results were interpreted as resistant (R), intermediate (I) and sensitive (S) and bacterial isolates resistant to more than three classes if a drug was classified as multidrug-resistant (MDR) [35, 36]. These antibacterial discs were selected based on the pathogen, local availability, commonly prescribed antibiotics in the study area and CLSI, 2022 recommendations.

The following antimicrobials (Oxoid, LTD, UK) were tested for Gram-negative bacteria: ampicillin (AMP, 10μg), amoxicillin-clavulanic acid (AMC, 30 μg), ceftriaxone (CRO, 30μg), ceftazidime (CAZ, 30μg), amikacin (AMK, 30μg), gentamicin (GEN, 10 μg), meropenem (MEM, 10μg) ciprofloxacin (CIP, 5μg), trimethoprim/sulfamethoxazole (SXT, 1.25/23.75μg), tetracycline (TCY, 30μg) and chloramphenicol (CHL, 30μg).

For Gram-positive bacteria: ciprofloxacin (CIP, 5μg), gentamicin (GEN, 10μg), ceftriaxone (CRO, 5μg), azithromycin (AZM, 15μg) clindamycin (CLD, 2μg) trimethoprim/sulfamethoxazole (SXT,

1.25/23.75μg), penicillin (P, 10 units), cefoxitin (FOX, 30μg), chloramphenicol (CHL, 30μg) and doxycycline (DOX, 30μg) were tested [35].

Data quality assurance

A structured questionnaire was prepared in a clear and precise way. The validation of the questionnaire was checked by doing a pre-test on 5% of the total sample size (19 clients) at Asrade Zewudie Memorial Hospital. Data collectors were trained and standard operating procedures (SOPs), as well as manufacturer's instructions, were strictly followed during specimen collection, transportation and processing. Preparation of all culture media, and their sterility was checked by overnight incubation of 5% of the batch at 37°C and observing for growth. Any growth in the culture medium was rejected, and replaced by new sterile batch. All prepared media and antibiotics were checked by inoculating standard reference strains, *S. aureus* (ATCC® 29213), *E. coli* (ATCC® 25922), *P. aeruginosa* (ATCC® 27853) and *S. pneumonia* (ACCT®49619) as quality control during the study period.

Data processing and analysis

Data were entered by EPI-data -version 4.6 and exported to Statistical Package for Social Science (SPSS) program version 25 for analysis. Bivariable logistic regression was carried out to identify the associated factors with bacterial profile of gowns, stethoscopes, and mobile phones. Multivariate logistic regression analysis was fitted to variables that had a *P*-value of less than or equal to 0.25 in bivariable logistic regression. Finally, multivariate logistic regression analysis was performed for adjusted odds ratio (AOR) with 95% confidence intervals. A *P*-value of ≤0.05 with 95%CI was considered statistically significant. The Hosmer and Lemeshow goodness of fit test was used for model fitness. Antimicrobial susceptibility tests were analyzed by using WHONET software.

Ethical considerations

The data was collected after ethical clearance was obtained from the Institutional Research Ethics Review Committee (IRERC) of College of Medicine and Health Sciences, Debre Markos University (Reference number: HSC/RCS/127/11/12). A supporting letter was written from the College of Medicine and Health Sciences to DMCSH. Then, a written permission paper was obtained from DMCSH before data collection. All the study participants were informed about the aim of the research and written consent was obtained. Participants were free to continue or withdraw from the study. Confidentiality of the results were maintained.

RESULTS

Socio-demographic characteristics of healthcare professionals

A total of 376 inanimate objects were swabbed from 191 healthcare professionals. Of these (63.9%; 122/191) were from male HCPs. The mean age of the participants was 30.43 +/- 4.809 and (range 20 to 45) years. More than fourty percent (42.9%; 82/191) of the HCPs were between the age of 30 -34 years. Of the total HCPs who participated in this study, most of them (59.2%; 113/191) had less than 5 years of service. With respect to field of specialization, most of the participants (25.7%; 49/191) were nurse professionals. Regarding working wards (31.9%, 61/192), (16.8%; 32/191), and (9.9%; 19/191) HCPs' inanimate objects were from OPD, GYN/Obs and surgical ward, respectively. From the total inanimate objects, (33.5%, 126/376), (33.5%; 126/376), and (33%; 124/376) were mobile phones, gowns and stethoscopes, respectively (**Table 1**).

Table 1: Socio-demographic characteristic of HCPs from inanimate objects used by healthcare professionals at DMCSH, Northwest Ethiopia 2023

**Variables**		**Frequency (N)**	**Percentage (%)**
Sex	Male	122	63.9
	Female	69	36.1
Age(in years)	20-24	17	8.9
	25-29	56	29.3
	30-34	82	42.9
	>35	36	18.8
Year of service	<5 year	113	59.2
	5-10 year	55	28.8
	>10 year	23	12.0
Field of specialization	Nurse	49	25.7
	Lab. Professional	10	5.2
	Pharm. Professional	11	5.8
	Medical Doctor	40	20.9
	Midwifery	11	5.8
	Intern students	61	31.9
	Others*	9	4.7
Level of education	Diploma	11	5.8
	BSc	74	38.7
	MD	26	13.6
	Med. intern students	61	31.9
	Specialist	19	9.9
Wards	OPDs	61	31.9
	Pediatrics	15	7.9
	Gynecology/ Obs.	32	16.8
	ICU & NICU	15	7.9
	Laboratory unit	10	5.2
	OR	10	5.2
	Surgical	19	9.9
	Medical	18	9.4
	Pharmacy unit	11	5.8
Inanimate object	Gown	126	33.5
	Mobile phone	126	33.5
	Stethoscope	124	33.0

*Physiotherapy, X-ray technicians, anesthesia, ophthalmology profession, psychiatry and dentistry; MD: Medical Doctor; ICU&NICU: Intensive care unit and Neonatal intensive care unit; OPD: Outpatient department; OR: Operation room

Hygiene-related practice of healthcare professionals

The overall HCPs' practice towards regular hand washing and disinfection of their inanimate objects were presented in Table 2. The majority of the HCPs did not regularly wash their hands after touching

a patient (79.6%; 152/191). Moreover, (61.1%; 77/126) of the HCPs did not regularly disinfect their mobile phones. More than three fourth of the participants (71.4%; 91/126) used their mobile phones at bedside for medical information.

Table 2: Hygiene-related practice of HCPs from inanimate objects used by healthcare professionals at DMCSH, Northwest Ethiopia 2023

**Variables**	**Frequency**	
	**Yes N(%)**	**No N(%)**
Regular hand washing before attending the patient	135(70.7)	56(29.3)
Regular hand washing after touching a patient	39(20.4)	152(79.6)
Regular use antiseptic for hands	113(59.2)	78(40.8)
Regular hand washing after body fluid exposure	179(93.7)	12(6.3)
Regular hand washing before and after clean/aseptic procedure	153(80.1)	38(19.9)
Regular disinfection of mobile phone	49(38.9)	77(61.1)
Use of mobile phone at bed side for medical information	90(71.4)	36(28.6)
Wash hands after using a mobile phone in the hospital	35(27.8)	91(72.2)
Carry mobile phone with patient care material	82(65.1)	44(34.9)
Answer phone calls while attending patients	62(49.2)	64(50.8)
Regularly disinfection of stethoscope after examining each patient	43(34.7)	81(65.3)
Share stethoscope	34(27.4)	90(72.6)
Use laundry for your gown/ clean your gown regularly	91(72.2))	35(27.8)
Think that gown, mobile phones and stethoscope can carry bacteria	182(95.3)	9(4.7)
Taking any training in infection prevention	88(46.1)	103(53.9)
Infection prevention manual in working area	153(80.1)	38(19.9)

Prevalence of bacterial contamination

Overall, 60.1% (226/376) (95%CI: 55.1- 65) inanimate objects were contaminated with different bacteria. Mobile phones had the highest percentage of bacterial contamination (65.1%; 82/126), followed by gowns (60.3%; 76/126) and stethoscopes (54.8%; 68/124). Study participants (70.2%, 40/57 from mobile phone and (66.1%; 39/59) from gown had less than 5 years of service, whereas, (63.0%; 17/27) from stethoscope had 5-10 years of service were a high rate of bacterial

contamination. By profession, the contamination rate of inanimate object was (90%; 9/10) laboratory professionals' mobile phone, (80%; 12/13) intern students' gown and (73.3%; 11/20) nurses' stethoscope. The highest frequency of inanimate object contamination with bacteria was observed among HCPs working in intensive care unit and neonatal care unit (ICU &NICU) (87.5%; 7/8/) stethoscope, and (83.3; 10/12) gown and (90.0%; 9/10) mobile phone from laboratory unit (**Table 3**).

Table 2: Bacterial contamination of inanimate objects used by healthcare professionals at DMCSH, Northwest Ethiopia 2023

**Characteristics**		**Contamination status**					
		**Gown**		**Mobile phone**		**Stethoscope**	
		**Contaminated N (%)**	**Non-contaminated N(%)**	**Contaminated N (%)**	**Non-contaminated N(%)**	**Contaminated N (%)**	**Non-contaminated N(%)**
Sex	Male	49(60.5)	32(39.5)	52(64.2)	29(35.8)	46(55.4)	37(44.6)
	Female	27(60.0)	18(40.0)	30(66.7)	15(33.3)	22(53.7)	19(46.3)
Age (in years)	20-24	7(41.2)	10(58.8)	10(55.6)	8(44.4)	4(66.7)	2(33.3)
	25-29	22(64.7)	12(35.3)	19(54.3)	16(45.7)	22(56.4)	17(43.6)
	30-34	33(67.3)	16(32.7)	35(71.4)	14(28.6)	25(45.5)	30(54.5)
	>35	14(53.8(	12(46.2)	18(75.0)	6(25.0)	17(70.8)	7(29.2)
Year of service	<5 year	39(66.1)	20(33.9)	40(70.2)	17(29.8)	47(52.2)	43(47.8)
	5-10 year	27(57.4)	20(42.6)	29(60.4)	19(39.6)	17(63)	10(37)
	>10 year	10(50.0)	10(50.0)	13(61.9)	8(38.1)	4(57.1)	3(42.9)
level of education	Diploma	6(54.5)	5(45.5)	6(54.5)	5(45.5)	0	0
	BSc	39(56.5)	30(43.5)	42(60.9)	27(39.1)	13(59.1)	9(40.9)
	MD	10(62.5)	6(37.5)	11(68.8)	5(31.3)	12(48.0)	13(52.0)
	Med. intern students	12(80.0)	3(20.0)	11(73.3)	4(26.7)	30(49.2)	31(50.8)
	Specialist	9(60.0)	6(40.0)	12(80.0)	3(20.0)	13(81.3)	3(18.8)
Field of specialization	Nurses	26(57.8)	19(42.2)	26(57.8)	19(42.2)	11(73.3)	9(26.7)
	Lab. Professionals	6(60.0)	4(40.0)	9(90.0)	1(10.0)	0	0
	Pharm. professionals	4(36.4)	7(63.6)	5(45.5)	6(54.5)	0	0
	Doctor	17(65.4)	9(34.6)	20(76.9)	6(23.1)	23(59.0)	16(41.0)
	Midwifery	7(70.0)	3(30.0)	5(50.0)	5(50.0)	2(50.0)	2(50.0)
	Intern students	12(80.0)	3(20.0)	11(73.3)	4(26.7)	30(49.2)	31(50.8)
	Others	4(44.4)	5(55.6)	6(66.7)	3(33.3)	2(40)	3(60)
Wards	OPDs	21(50)	21(50)	25(59.5)	17(40.5)	22{55)	18(45)

Pediatrics	3(50)	3(50)	1(16.7)	5(83.3)	6(42.9)	8(57.1)
Gynecology/ Obs.	14(82.4)	3(17.6)	12(70.6)	5(29.4)	13(52)	12(48)
ICU & NICU	10(83.3)	2(16.7)	9(75)	3(25)	7(87.5)	1(12.5)
Laboratory unit	6(60)	4(40)	9(90)	1(10)	0	0
OR	5(62.5)	3(37.5)	6(75)	2(25)	4(57.1)	3(42.9)
Surgical	6(54.5)	5(45.5)	7(63.6)	4(36.4)	8(53.3)	7(46.7)
Medical	7(77.8)	2(22.2)	8(88.9)	1(11.1)	8(53.3)	7(53.3)
Pharmacy unit	4(36.4)	7(63.6)	5(45.5)	6(54.5)	0	0

OPD: Outpatient department, ICU & NICU: Intensive care unit&neonatal intensive care unit, OR: Operation room, MD: Medical Doctor, BSc: Bachelor of Science

Bacterial isolates from HCPs inanimate objects

Out of 376 swab samples processed, (61.7%; 232/376) bacteria were isolated. Only six inanimate objects showed co-infection with two bacterial species. From the total bacterial isolates (63.4%; 147/232) and (36.6%, 85/232) were Gram-positive and Gram-negative bacteria, respectively. Overall, *S. aureus* was the predominant isolate accounting (22.1%, 83/376) followed by *S. epidermidis* (17.0%, 64/376), *E. coli* (8.8%, 33/376) and *P. aeruginosa* (4.9%; 18/376). *S. aureus* was the predominant isolate from gown 39.2% (31/83) and from mobile phone (41%; 34/83). On the other hand, *S. epidermidis* was the most frequent isolate from stethoscope (35.9%; 23/64) (**Fig 2**).

Figure 2: Proportion of bacterial isolates from healthcare professionals' inanimate objects used by healthcare professionals at DMCSH, Northwest Ethiopia, 2023

Factors associated with contamination of healthcare professionals' inanimate objects

From bi-variable analysis age, level of education, working ward, regularly washing hands before attending patient, hand washing after touching patient, regular use of antiseptic for hands, regular hand washing before and after clean/aseptic procedure, regular disinfection of mobile phone, use of mobile phone at bedside to medical information, carry mobile phone with patient care material, regular disinfection of stethoscope after each patient, share stethoscope, use laundry for gown/ clean gown regularly, infection prevention training and presence of infection prevention manual were candidates for multivariate analysis ($P \leq 0.25$).

In multivariate analysis, inanimate objects of HCPs from Gyn/Obs ward (AOR: 8.69; 95%CI: 1.09-69.41, *P*=0.041), regular disinfection of mobile phone (AOR: 2.69; 95%CI: 1.15-6.25; *P*=0.021), carry mobile phone with patient care material, (AOR: 2.72; 95%CI: 1.18-6.29, *P*=0.019), regular disinfection of stethoscope after examining each patient (AOR: 3.06, 95% CI: 1.23-7.59; *P*=0.016) and no taking infection prevention training (AOR: 3.91; 95%CI:1.71-8.93; *P*=0.001) were associated with bacterial contamination of HCPs inanimate objects (**Table 4 and 5**).

Table 4: Socio-demographic factors associated with contamination of HCPs inanimate objects used by HCPs at Debre Markos Comprehensive Specialized Hospital, Northwest Ethiopia 2023

**Variables**		**Inanimate object contamination**		**COR(95% CI)**	**P-value**	**AOR(95%CI)**	**P-value**
		**Yes (%)**	**No (%)**				
Age	20-24	10(55.6)	8(44.4)	0.42(0.11-1.55)	0.190	0.61(0.09-3.71)	0.591
	25-29	19(54.3)	16(45.7)	0.39(0.13-1.24)	0.111	0.36(0.07-2.02)	0.25
	30-34	35(71.4)	14(28.6)	0.83(0.27-2.54)	0.748	1.31(0.266-6.46)	0.74
	>35	18(75.0)	6(25.0)	1			
Year of service	< 5 years	39(66.1)	20(33.9)	1.95(0.69-5.46)	0.203	0.89(0.24-3.42)	0.876
	5-9 years	27(57.4)	20(42.6)	1.35(0.47-3.86)	0.575	0.77(0.196-2.99)	0.702
	>10 years	10(50.0)	10(50.0)	1			
Level of education	Diploma	6(54.5)	5(45.5)	0.80(0.17-3.86)	0.781	1.45(0.17-9.72)	0.701
	BSc	39(56.5))	30(43.5)	0.87(0.28-2.70)	0.805	0.67(0.18-2.52)	0.550
	MD	10(62.5)	6(37.5)	1.11(0.26-4.72)	0.886	1.74(0.32-9.45)	0.524
	Med. Intern	12(80.0)	3(20.0)	2.67(0.52-13.66)	0.239	1.82(0.24-13.64)	0.558
	Specialist	9(60.0)	6(40.0)	1			
Working ward	OPD	21(50.0)	21(50.0_	1.75(0.45-6.88)	0.423	1.85(0.37-9.27)	0.454
	Pediatrics	3(50.0)	3(50.0)	1.75(0.23-13.16)	0.587	1.65(0.16-17.22)	0.675
	GYN/OBS	14(82.4)	3(17.6)	8.17(1.42-47.02)	0.019	8.69(1.09-69.41)	0.041*
	ICU & NICU	10(83.3)	2(16.7)	8.75(1.24-61.68)	0.029	8.82(0.95-81.45)	0.055
	Laboratory unit	6(60.0)	4(40.0)	2.63(0.45-15.31)	0.283	1.84(0.26-12.91)	0.539
	OR	5(62.5)	3(37.5)	2.92(0.44-19.23)	0.266	3.19(0.34-29.74)	0.309
	Surgical ward	6(54.5)	5(45.5)	2.10(0.38-11.59)	0.395	2.29(0.29-17.75)	0.427
	Medical ward	7(77.8)	2(22.7)	6.13(0.83-45.02)	0.075	4.37(0.42-45.18)	0.216
	Pharm. Unit	4(36.4)	7(63.6)	1			

Key: BSc: Bachelor of Science, MD: Medical doctor, OR: Operation theatre, OPD: Outpatient department, GYN/OBS: Gynecology/obstetrics, ICU&NICU: Intensive care unit and Neonatal Intensive care unit

Table 5: Hygiene-related factors associated with contamination of HCPs inanimate objects used by HCPs at Debre Markos Comprehensive Specialized Hospital, Northwest Ethiopia 2023

**Variables**		**Inanimate object**		**COR (95% CI)**	**P-value**	**AOR(95%CI)**	**P-value**
		**Yes (%)**	**No (%)**				
Hand washing before attending patient	Yes	54(61.4)	34(38.6)	1			
	No	14(38.9)	22(61.1)	0.40(0.18-0.89)	0.024	0.43(0.17-1.11)	0.081
Hand washing after touching patient	Yes	20(57.1)	15(42.9)	1			
	No	62(68.1)	29(31.9)	1.60(0.72-3.57	0.248	1.56(0.47-5.19)	0.469
Regularly use antiseptic for hands	Yes	43(62.3)	26(37.7)	1			
	No	25(45.5)	30(54.5)	0.50(.25-1.04)	0.062	0.43(.18-1.05)	0.065
Regular hand washing before and after clean/aseptic procedure	Yes	56(60.9)	36(39.1)	1			
	No	26(76.5)	8(23.5)	2.09(.85-5.12)	0.107	2.14(0.63-7.31)	.0.223
Regular disinfection of mobile phone	Yes	23(46.9)	26(53.1)	1			
	No	59(76.6)	18(23.4)	3.71(1.715-8.01)	0.001	2.69(1.15-6.25)	0.021*
Use mobile phone for medical information	Yes	54(60.0)	36(40.0)	0.43(0.18-1.05)	0.063	0.63(0.15-2.69)	0.534
	No	28(77.8)	8(22.2)	1			
Carry mobile phone with patient care material	Yes	48(77.4)	14(22.6)	3.03 (1.39-6.55)	0.005	2.72(1.18-6.29)	0.019*
	No	34(53.1)	30(46.9)	1			
Use laundry for your gown/ clean your gown regularly	Yes	51(56.0)	40(44.0)	1			
	No	25(71.4)	10(28.6)	1.96(0.85-4.55)	0.117	2.45(0.99-6.07)	0.052
Regular disinfection of stethoscope after examining each patient	Yes	16(37.2)	27(62.8)	1			
	No	52(64.2)	29(35.8)	3.03(1.41-6.52)	0.005	3.06(1.23-7.59)	0.016*
Share stethoscope	Yes	12(35.3)	22(64.7)	0.33 (0.15-0.75)	0.008	0.56(0.18-1.75)	0.317
	No	56(62.2)	34(37.8)	1			
Infection prevention training	Yes	20(35.1)	37(64.9)	1			
	No	48(71.6)	19(28.4)	4.67(2.19-9.99)	0.000	3.91(1.71-8.93)	0.001*
Infection prevention manual	Yes	50(56.2)	39(43.8)	1			
	No	26(70.3)	11(29.7)	1.84(0.81-4.19)	0.144	1.46(0.56-3.81)	0.443

Antimicrobial susceptibility pattern

Among Gram-negative bacterial isolates, *E. coli* (n=33) was highly resistance to ampicillin and (97%, 32/33) and amoxacilline-clavunic acid and trimethoprim-sulfamethoxazole each accounts (93.9%, 31/33). While, meropenem (100%) and ciprofloxacin (90.9 %) were sensitive against *E. coli* isolates. On the other hand, *Klebsiella* sppecies showed high resistance rate to ampicillin, trimethoprim-sulfamethoxazole and amoxacilline-clavunic acid each accounts (92.3%; 12/13). However, *Klebsiella* spp. showed lower resistance rate against meropenem, ciprofloxacin and gentamicin, (0%, 0/13), (15.4%, 2/13) and (30.8%, 4/13), respectively. Similarly, *P. aeruginosa* showed a higher resistance rate against piperacillin (94.4%,17/18), cefetazidim (72.2%, 13/18) and ciprofloxacin (66.6%, 12/18) and lower resistance rate to meropenem (11.1%; 2/18) (**Table 6**).

Table 6: Antimicrobial susceptibility patterns of Gram-negative isolates from inanimate objects used by healthcare professionals at DMCSH, Northwest Ethiopia 2023

**Gram-negative bacteria**		**Antimicrobials with susceptibility profile N (%)**										
			**AMK**	**CAZ**	**PIP**	**MEM**	**GEN**	**CIP**	**TOB**			
P. aeruginosa (n=18)	S		14(77.8)	5(27.8)	0	16(88.9)	8(44.4)	6(33.3)	9(50)			
	I		0	0	1(5.6)	0	0		0			
	R		4(22.2)	13(72.2)	17(94.4)	2(11.1)	10(55.6)	12(66.7)	9(50)			
		**AMP**	**AMC**	**CXM**	**CAZ**	**CRO**	**MEM**	**GEN**	**CIP**	**SXT**	**CHL**	**TCY**
Klebsiella spp. (n=13)	S	0	1(7.7)	5(38.5)	7(53.8)	6(46.2)	13(100)	9(69.2)	11(84.6)	0	7(53.8)	5(38.5)
	I	1(7.7	0	1(7.7)	0	1(7.7)	0	0	0	1(7.7)	0	0
	R	12(92.3)	12(92.3%)	7(53.8)	6(46.2)	6(46.2)	0	4(30.8)	2(15.4)	12(92.3)	6(46.2))	8(61.5)
Enterobacter spp. (n=8)	S	1(12.5)	1(12.5)	4(50)	5(62.5)	4(50)	8(100)	7(87.5)	7(87.5)	0	5(62.5)	4(50)
	I	0	1(12.5)	0	0	0	0	0	0	1(12.5)	0	0
	R	7(87.5)	6(75)	4(50)	3(37.5)	4(50)	0	1(12.5)	1(12.5)	7(87.5)	3(37.5)	4(50)
Citrobacter spp. (n=8)	S	0	0	2(25)	5(62.5)	5(62.5)	8(100)	5(62.5)	7(87.5)	0	3(37.5)	1(12.5)
	I	1(12.5)	1(12.5)		1(12.5)	0	0	0	0	1(12.5)	0	1(12.5)
	R	7(87.5)	7(87.5)	6(75)	2(25)	3(37.3)	0	3(37.3)	1(12.5)	7(87.5)	5(62.5)	6(75)
Providencia spp. (n=3)	S	0	1(33.3)	1(33.3)	2(66.7)	2(66.7)	3(100)	3(100)	3(100)	0	1(33.3)	1(33.3)
	I	1(33.3)	0	1(33.3)	0	1(33.3)	0	0	0	1(33.3)	0	0
	R	2(66.7)	2(66.6)	1(33.3))	1(33.3)	0	0	0	0	2(66.7)	2(66.7)	2(66.7)
Serratia (n=2)	S	0	0	1(50)	1(50)	2(100)	2(100)	1(50)	2(100)	1(50)	01(50)	1(50)
	I	0	1(50)	0	1(50)	0	0	0	0	0	0	0
	R	2(100)	1(50)	1(50)	0	0	0	1(50)	0	1(50)	1(50)	1(50)
E.coli (n=33)	S	0	1(3)	13(39.4)	24(72.2)	19(57.6)	33(100)	27(81.8)	30(90.9)	0	16(48.5)	10(30.3)
	I	1(3)	1(3)	1(3)	1(3)	0	0	0	1(3)	2(6.1)	0	1(3)
	R	32(97)	31(93.9)	19(57.6)	8(24.2)	14(42.4)	0	6(18.2)	2(6.1)	31(93.4)	17(51.5)	22(66.7)

S. aureus showed high sensitivity to clindamycin (91.6%, 76/83) and gentamycin (84.3%, 70/83). However, it was highly resistant to penicillin (97.6%, 81/83), trimethoprim-sulfamethoxazole (67.5%, 56/83), azithromycin (50.6%, 42/83) and cefoxitin (36.1%, 30/83).

On the other hand, *S. epidermidis* was resistant to penicillin (98.4%, 63/64), trimethoprim-sulfamethoxazole (65.6%, 42/64), and doxycycline (48.4%, 31/64). Based on cefoxitin resistance (36.1%, 30/83) was methicillin-resistant *S. aureus* (MRSA) (**Table 7**).

Table 7: Antimicrobial susceptibility patterns of Gram-positive isolates from inanimate objects used by healthcare professionals at DMCSH, Northwest Ethiopia 2023

Gram-positive bacteria	AMP	**Antimicrobials susceptibility profile N (%)**									
		**PEN**	**FOX**	**OXA**	**GEN**	**CIP**	**SXT**	**CLN**	**AZM**	**CHL**	**DOX**
S. aureus (n=83)	S	2(7.2)	53(63.9)	ND	70(84.3)	60(72.3)	18(21.7)	76(91.6)	35(42.2)	58(69.9)	44(53)
	I	-	-	ND	1(1.2)	3(3.6)	6(7.2)	1(1.2)	6(7.2)	2(2.4)	8(9.6)
	R	81(97.6)	30(36.1)	ND	12(14.5)	20(24.1)	56(67.5)	6(7.2)	42(50.6)	23(27.7)	31(37.3)
S. epidermidis	S	1(1.6)	ND	40(62.5)	52(81.3)	36(56.3)	20(31.2)	56(87.5)	29(45.3)	48(75)	27(42.2)
(n=64)	I	-	ND	-	0	7(10.9)	2(3.1)	0	4(6.3)	0	6(9.3)
	R	63(98,4)	ND	24(37.5)	12(18.9)	21(32.8)	42(65.6)	8(12.5)	31(48.4)	16(25)	31(48.4)

Key: AMP: Ampicillin, CRO: Ceftriaxone, CHL: Chloramphenicol PEN: Penicillin, CIP: Ciprofloxacin, TCY: Tetracycline, CN: Gentamicin, FOX: Cefoxitin, SXT: Cotrimoxazole, CLN: Clindamycin, DOX: Doxycycline and AMC: Amoxacilline-clavunic acid, CXM: Cefuroxime, CAZ: Ceftazidim, MEM: Meropenem, FEP: Cefepime AZM: Azithromycin, OXA: Oxacilline, ND: Not done

Multidrug resistance profiles of the isolates

Multidrug resistance among Gram-positive and Gram-negative bacteria isolates were (65.3%; 96/147) and (75.3%; 64/85), respectively with over all MDR (69%, 160/232). Among all bacteria isolates *E. coli* (84.8%; 28/33) predominantly showed MDR followed by *Klebsiella* species (76.9%; 10/13), *P. aeruginosa* (72.2%;13/18), *S. epidermidis* (67.2%; 43/64), and *S. aureus* (63.9%, 53/83). From Gram-negative bacteria, *E. coli* showed MDR among 28 isolates (84.8%, 28/33) isolates. Similarly, *Klebsiella* species showed MDR among (76.9%; 10/13) (**Table 8**). *P. aeruginosa* were resistance to three or more antimicrobials among 13 isolates (72.2%; 13/18) (**Supplementary 1**). Of Gram-positive isolates, *S. epidermidis* was multidrug resistance among 46 isolates (71.8%; 46/64). Similarly, *S. aureus* was MDR among 53 isolates (63.8%; 53/83) isolates (**Suplementary 2**).

Table 8: Multidrug resistance pattern of Gram-negative isolates from inanimate objects used by healthcare professionals at DMCSH, Northwest Ethiopia 2023

**Name of Antimicrobial**	**No of classes non susceptible**	***E. coli* (n=33) n (%)**	***Klebsiella* spp. (n=13)**	***Enterobacter* spp. (n=8) n (%)**	***Citrobacter* spp. (n=8) n (%)**	***Providencia* spp. (N=3) n (%)**	***Serratia* spp. (n=2) n (%)**
None						1(33.3)	
AMP							1(50)
AMP, AMC	2	2(6.1)	1(7.7)	1(12.5)			
AMP, SXT		2(6.1)	1(7.7)	1(12.5)		-	
AMC, SXT		1(3)	1(7.7)	1(12.5)	1(12.5)	-	-
AMP,TCY		-			1(12.5)		
AMP, AMC, SXT	3	4(12.1)	-			-	-
AMP, AMC, CXM, CIP, SXT	5	2(6.1)	1(7.7)				
AMP, AMC, SXT,CXM, CHL, TCY	6	2(6.1)	-			1(33.3)	-
AMP, AMC, SXT, GEN, CHL, TCY		5(15.2)	1(7.7)	1(12.5)		-	-
AMP,AMC, GEN, CRO,SXT,CXM					1(12.5)		
AMP, AMC, SXT,CXM, GEN, TCY		1(3)	-			-	-
AMP, AMC, SXT, GEN, CHL, TCY,CXM					2(25)		
AMP, AMC, CXM, SXT, TCY,CAZ,CRO		4(12.1)	2(15.4)	2(25)	2(25)		
AMP, AMC, CXM, CRO, SXT, CHL, TCY	7	6(18.2)				1(33.3)	-
AMP, AMC, CXM, CAZ, CRO, SXT, CHL			2(15.4)	1(12.5)		-	-
AMP, AMC, GEN, SXT, CHL, TCY ,CXM						-	1(50)
AMP, AMC, CXM, CAZ, CRO, SXT,TCY,CHL		4(12.1)	2(15.4)			-	-
AMP,AMC, GEN, CIP, CHL, TCY,SXT,CXM	8	-	2(15.4)		1(12.5)		
AMP, AMC, CXM, CRO, SXT, CHL,CIP, TCY		-		1(12.5)			
Total		33(100)	13(100)	8(100)	8(100)	3(100)	2(100)

Key: AMP: Ampicillin, CRO: Ceftriaxone, CHL: Chloramphenicol PEN: Penicillin, CIP: Ciprofloxacin, TCY: Tetracycline, CN: Gentamicin, FOX: Cefoxitin, SXT: Cotrimoxazole, CLN: Clindamycin, DOX: Doxycycline and AMC: Amoxacilline-clavunic acid, CXM: Cefuroxime, CAZ: Ceftazidim, MEM: Meropenem, FEP: Cefepime AZM: Azithromycin, OXA: Oxacilline

Discussion

Inanimate objects including stethoscopes, mobile phones, and gowns are essential materials in HCPs' day-to-day activities throughout the world. These inanimate objects are used by healthcare professionals' are thought to be possible sources of diseases related to healthcare because they collect dangerous germs and spread the infection to others. They are also particularly susceptible to bacterial contamination from the hospital context [37].

The overall prevalence of bacterial contamination of HCPs' inanimate objects was (60.1%; 95%CI: 55.1% to 65%) which was in line with result reported in Bahir Dar, Ethiopia (57.6%) [9]. However, the current study was lower than a study conducted in Harar, Ethiopia (94.2%) [38], Nigeria (77.7%) [39], Tanzania (73.3%) [21] and another study from Nigeria (65.5%) [40]. On the other hand, this is higher than result reported in Pakistan (43%) [41], Saudi Arabia (30%) [42] and Rajshahi 19% [43]. The observed variation may have resulted from variations in the adherence to hygiene-related practices, regular hand washing practices, HCPs' awareness of the role of inanimate objects play in microbial transmission, variations in the regular laundry gown use habits, and trends of inanimate objects use and storage.

In the present study, the prevalence of bacterial contamination of HCPs' mobile phones was 65.1% (56.1%-73.4%, 95% CI), which was similar to a study conducted in Addis Ababa, Ethiopia (62%) [44], Jimma (71.2%) [45]. However, it was lower compared to a research conducted in Gondar, Ethiopia (98%) [46], India (100%) [47], Italia (100%) [48], China (95.5%) [49] and in Sana'a city, Yemen (70%) [50]. On the other hand, the current study was higher compared to previous report from Saudi Arabia (36.9%) [51] and Egypt (46.3%) [52]. The differences in the study HCPs adherence to infection prevention, mobile phone usage patterns, variation in regular disinfecting of mobile phones, and personal behaviors, such as picking noses and touching their phones, could all be contributing factors to the variation in the contamination.

In this study, the prevalence of bacterial contamination of HCPs' gown was 60.3% (51.6%-68.5%, 95%CI). It was in line with a study conducted in Bahir Dar (55.7%) [9]. However, it was lower than study conducted in Tanzania (73.3%) [21] and Nigeria (77.7%) [39]. But higher than study conducted in Nepal (50%) [53] and America (0-16%) [54]. This discrepancy might be due to differences in the use of regular laundry gowns (practice of cleaning gowns).

The prevalence of bacterial contamination of HCPs stethoscopes was 54.8% (45.7-63.8%, 95%CI). This was in line with study conducted in Addis Ababa, Ethiopia (53.8%) [55] and India (56%) [24]. However, this was lower than study conducted in Gurage, Ethiopia (69.2%)[56], Sweden (86%) [23] and India (79%) [57]. But, this study was higher than study conducted in Saudi Arabia (30%) [42] and Bangladesh (19%) [43]. The observed differences might be the result of variations in stethoscope routine disinfecting practice.

By profession, the contamination rate of inanimate objects was higher in laboratory professionals' mobile phone, intern students' gown and nurses' stethoscope. This was agree with result reported from Bahir Dar medical laboratory professionals, nurses and medical intern students, respectively [9]. The highest frequency of inanimate object contamination with bacteria was observed among HCPs' mobile phone working in laboratory unit, and HCPs' stethoscope and gown in NICU/ ICU. This was agree with result reported from Egypt laboratory unit [13], Jimma ICU, Ethiopia [58].

From the total bacterial isolate (63.4%; 147/232) and (36.6%, 85/232) were Gram-positive and Gram-negative bacteria, respectively. It was supported by previous studies at Bahir Dar (84.9%, 15.1%), and [9], Jimma (78.9%, 21.1%), respectively [46]. The reason for high prevalence of Gram-positive bacteria, direct contact of inanimate objects to human skin flora which contains mostly Gram-positive bacteria, gram positive bacteria alive for a long period or Gram-positive can tolerate inanimate objects for a long period of time, but Gram-negative bacteria short lifespan.

In the present study *S. aureus* (22.1%, 95% CI: 17.9-26.6%), *S. epidermidis* (17.0%, 95%CI: 13.4-21.2%), *E. coli* (8.8%, 95%CI: 6.1-12.1) and *P. aeruginosa* (4.9%; 95%CI: 2.86-7.46) were the most common isolates. The majority of earlier research, conducted in Ethiopia [38, 46] and outside the country, in Nigeria and Zagazig [39, 52] reported comparable bacterial isolates with varying rates of isolation. Similarly, in this study, *S. aureus* isolates were lower than study conducted in Asella, Ethiopia (45%) [59], and Karachi (70%) [10]. *S. aureus* is an opportunistic pathogen that frequently colonizes human skin as well as respiratory tract and might be transmitted to HCPs' inanimate objects via sneezing and coughing. However, if it gets passed the skin's protective layer, it can cause a range of acute and chronic infections, pyogenic and systemic infections. respiratory tract and might be transmitted to the inanimate objects via coughing and sneezing.

S. epidermidis was the second most frequent isolates in the current study. However, the magnitude of the present study (17.0%, 95%CI:13.4-21.2%), this was in line with previous study done in Debre Berhan, Ethiopia (16.7%) [60], Harar, Ethiopia (14.4%) [38] and Benegal **(**14.47%) [61]. This was lower than the previous study done in Bahir Dar (44%) [9], and Jimma, Ethiopia (60.6%) [45]. This discrepancy might be due to differences in inanimate objects decontamination practice, hygiene practice and regular hand washing with antiseptic solutions in clinical settings.

From Gram-negative bacteria, *E. coli* (8.8%, 33/376; 95%CI: 6.1-12.1%) was the most predominant bacteria followed by *P. aeruginosa* (4.9%; 18/376; 95%CI: 2.86-7.46%). This was in line with study conducted in Gondar (6.8%) *E. coli* [46] and in Harare, Ethiopia (3.7%) [38]. However lower than result reported from Mizan-Tepi, Ethiopia (15.9%) *E. coli* [62], and (19.2%) *P. aeruginosa* [63]. But higher than reports from the previous study done Bahir Dar (0.24%) *P. aeruginosa* [31] and Benegal (1.32%) *E. coli* [61]. This discrepancy might be due to differences in inanimate objects decontamination, hygiene practice and regular hand washing with antiseptic solutions in clinical settings.

In the current study, highest risk of bacterial contaminations of inanimate objects were found in those healthcare professionals working at Gyn/Obs (AOR: 8.69; 95%CI: 1.09-69.41; *P*=0.041) compared to other working wards. This is comparable with previous studies conducted in Bahir Dar [9]. On contrary, highest risk of bacterial contamination was found in ICU from Debre Berhan, Ethiopia [60], at medical and surgical ward in Madda Walabu University Goba Referral Hospital, Ethiopia [64] and at the laboratory unit in Alexandria, Egypt [13]. The reasons for this discrepancy could include the following: variations in the study environment, variations in the wards' cleaning practices and insufficient adherence to infection control protocols, variations in the patient load, and variations in the frequency of HCWs' interactions with patients.

Different factors were associated with the contamination of inanimate objects. Healthcare professionals' mobile phones that did not regularly disinfect were 2.7 times more likely contaminated with bacteria compared to those who had more regularly cleaned their mobile phones. This was consistence with the previous result reported from Harar, Ethiopia [38] and Debre Berhan, Ethiopia [60]. Similarly, healthcare professionals who carry mobile phone with patient care materials were more than two times more likely contaminated with bacteria compared to those who did not carry mobile phone with patient care materials. This study was unsupported by study conducted in Harar,

Ethiopia [38] and Gondar, Ethiopia [46]. This difference might be due to differences in habit of mobile phone keeping.

In addition, healthcare professionals who did not regularly disinfect their stethoscopes after examining each patient were 3.1 times more likely contaminated with bacteria compared to those who had regularly cleaned their stethoscopes after examining each patient. This is in line with previous studies conducted in Medda Walabu University Goba Referral Hospital, Ethiopia [64], Eastern Ethiopia [65], Benegal [61], Nepal [66] and Bangladesh [43]. This study was unsupported by a study conducted in Saudi Arabia [42]. This difference might be the habit of keeping stethoscope and study setting.

Similarly, healthcare professionals who did not take any training in infection prevention were 3.9 times more likely contaminated with bacteria compared to those who had infection prevention training. This was agreed with a study reported from Bale-Goba, Ethiopia [64]. However, this disagrees with a report from Harar, Ethiopia [38]. Poor adherence to infection prevention practice in the current study area may be the cause of this disparity.

The most serious health risk is resistance to one or more antimicrobials [59, 67]. In this study, ampicillin was highly resistant to *E. coli* (97%; 95%CI; 82.5-99.8), *Klebsiella* spp. (92.3%; 95%CI; 62.1-99.6), and *Citrobacter* species (87.5%; 95%CI: 46.7-99.3). This was in line with previous study done in Harare (78.6), (75%) *E. coli* and *Citrobacter* species, respectively [38] and Mekelle (90.9) [68].

Trimethoprim-sulfamethoxazole was highly resistant to *E. coli* (93.9; 95%CI: 78.5-98.8), *Klebsiella* species (92.3%; 95%CI; 62.1-99.6) and *Citrobacter* species (87.5%; 95% CI:46.7-.99.3). This was consistent with previous studies done in Nigeria (90%,) *Klebsiella* spp. and (83.3%) *E. coli* [40], Harar (62.5%) *Citrobacter* species [38]. However, meropenem (0%) and ciprofloxacin (6.1%), for *E. coli* and (0%) and (15.4%) for *Klebsiella* species showed lower resistance rates, respectively which agreed with earlier research, conducted in Gondar 0% [69] and Harar 22.2% [38] reported lower rates of resistance, respectively.

In the current study *S. aureus* isolates showed high level of resistance to penicillin (97.6%, 95%CI: (82.5-99.9). This was consistent with previous study reported from Jimma, Ethiopia (88.2) [45] and Bahir Dar, Ethiopia (82.7%) [9]. However, it was relatively lower than a study in Zambia (100%)

[70]. But higher compared to Harar (61.3%) [38]. This might be due to differences in consumption of penicillin in the study area and they might have been common contamination sources by patients, the hospital-contaminated environments, or some other unidentified source (the first natural antibiotics that led to cover prescription, frequently used and overused of penicillin. While this study also showed highly sensitive to clindamycin (91.6%; 95%CI: 79.3-96.4)) and gentamycin (84.3% ; 95%CI: 78.9-99.9). This was in line with study conducted in Ethiopia, Bahir Dar (83%) gentamycin [9], Jimma (90.3%) clindamycin and (89.6%) gentamycin [58] and Nepal gentamycin (80.5) [66].

S. epidermidis was highly resistance to penicillin (98.4%, 95%CI: 82.9-99.9). This was in line with result reported from Turkey (88%) [71]. However, higher than previous study reported from Mizan (68.8%) [62], Bahir Dar, Ethiopia (82.7%) [9] and India (79.9%) [72]. This might be due to differences in the consumption of penicillin in the study area, the hospital-contaminated environments, empirical treatment protocols, use of antibacterial as a prophylactic, easily accessible non-prescription drugs (self-medication) and drug dosage.

The prevalence of methicillin-resistant *S. aureus* was found to be 36.1% (95%CI: 25.9-47.7) based on cefoxitin resistance. This was in line with the previous study conducted in Jimma (26.6%) [58]. However, lower than study conducted in Mizan-Tepi, Ethiopia (73.3%) [62], Pakistan (88.5%) [41], another study from Pakistan (58.4%) [73], and Zambia (48%) [70]. Similarly, this is higher compared to a study in Arba Minch, Ethiopia (19.1%) [74], India (3%) [72], and another study from India (6.4%) [75]. The observed variation in antimicrobial resistance when compared to previous studies could be attributed to various factors such as different bacterial strains, hospital environments, empirical treatment protocols, the use of antibiotics as preventive therapy, self-medication, drug dosage, and prolonged administration of common antibiotics [76].

P. aeruginosa showed higher resistance rate against piperacillin (94.4%, 95% CI: 70.6-99.9%), ceftazidim (72.2%; 95%CI: 46.9-89.3%) and ciprofloxacin (66.6%); 95%CI: 41.2-85.6%) and lower resistance rate to meropenem (11.1%). This was in line with study reported from Gondar 92.3% piperacillin [69], India (71.3%) ceftazidime [41], Harar 62.5% ciprofloxacin [38], Bengal (60%) ciprofloxacin [61], India (68.4%) ciprofloxacin [72] and (0%) meropenem [71]. However higher compared with study conducted in India (0%) piperacillin [72] and Hawasa (13%) ceftazidim [77]. But lower than reports from Nigeria (100%) ciprofloxacin [78] and Gondar (100%) cefepime and (92.3%) ciprofloxacin [69]. The observed variation in antimicrobial resistance when compared to

previous studies could be attributed to various factors such as different bacterial strains, hospital environments, empirical treatment protocols, the use of antibiotics as preventive therapy, the accessibility of certain drugs without a prescription, drug dosage, and prolonged administration of common antibiotics [76].

The overuse and unreasonable prescription of antibiotics may give rise to bacterial strains that are resistant to many drugs [38]. The overall MDR prevalence of the bacterial isolates from inanimate objects used by healthcare professionals in the current study was (69%, 95%CI: 62.1-74.5).This was in line with the previous study conducted in Arba Minch, Ethiopia (57.7%) [74] and in Harar, Ethiopia (69.9%) [38]. On the other hand, this finding was higher compared to a study in Debre Berhan, Ethiopia (42.9%) [60] and in Mizan-Tepi, Ethiopia (48.9%) [62]**.** On contrary, lower than the study conducted in Bahir Dar, Ethiopia (88.3%) [31]. The observed variation in antibiotic resistance may be caused by various factors such as distinct bacterial strains, hospital environments, empirical treatment protocols, use of antibacterial as a prophylactic, easily accessible nonprescription drugs (self-medication), drug dosage, and indiscriminate or prolonged use of common antibiotics [58, 79].

In this study, multidrug resistance rate among Gram-negative and Gram-positive bacteria isolates were (75.3%; 95%CI: 63.5-83%) and (65.8%; 95%CI:57.0-72.9%), respectively. This is disagree with study report from Bahir Dar, Ethiopia (88.3%, 94.7%), respectively [9]. Among Gram-positive isolates, *S. epidermidis* (67.2%; 95%CI: 54.3-78.4%) and *S. aureus* (63.9%; 95%CI: 52.6-74.1%) were showed multi-drug resistance. This is in line with result report from Harar, Ethiopia (71%, 66.1%) [38] and Debre Berhan, Ethiopia (53.8%, 64.3%), respectively [60]. However, this disagree with result reported from Bahir Dar, Ethiopia (86.4%, 88.9%), respectively [31]. This discrepancy might be due to differences in the isolation rate, inappropriate administration of antimicrobial drugs, and self-medication practice, in the study area the majority of antibiotic classes were utilized as alternative forms of treatment and might be cross contamination between patients and HCPs' inanimate objects [58].

From Gram-negative isolates *E. coli* (84.8%, (95%CI: 61.9-94.6%), *Klebsiella* species (76.9%, 95%CI: 42.5-92%), and *P. aeruginosa* (72.2, 95%CI; 35.7-89.5) were most predominantly showed MDR. This is comparable with reports from Harar, Ethiopia (86.7%) *Klebsiella* species and (87.5%) *P. aeruginosa*, respectively [38] and Bahir Dar Ethiopia (92.6%) *E. coli* [9]. However, this was

lower than result reported from Bahir Dar, Ethiopia (100%) *Klebsiella* species and (100%) *P. aeruginosa* [31]. But lower than result reported from Mizan-Tepi (28.6%), (53.8%) and (30%), respectively [62]. This discrepancy might be due to differences in the isolation rate, inappropriate administration of antimicrobial drugs, and self-medication practice, in the study area the majority of antibiotic classes were utilized as alternative forms of treatment [58]**.**

Conclusion

The results of the present study indicated that many of the inanimate objects of HCPs were contaminated with different bacteria The most common bacterial isolates were *S. aureus*, *S. epidermidis*, *E. coli* and *P. aeruginosa*. It also revealed that did not regularly disinfecting inanimate objects and those inanimate objects taken from gynecology/obstetrics ward were most likely to be contaminated. Moreover, those healthcare professionals who carried mobile phone with patient care materials were more than two times more likely to be contaminated with bacteria compared to those who did not carry mobile phone with patient care materials. Furthermore, those HCPs who did not take infection prevention training were more likely to be contaminated. Gram-positive isolates showed high level of resistance to penicillin and the majority of Gram-negative bacteria were resistant to ampicillin, trimethoprim/sulfamethoxazole, and amoxicillin-clavulanic. On the other hand, meropenem for Gram-negative and clindamycin for Gram-positive bacteria showed higher level of sensitivity. Among all bacterial isolates, *E. coli* was most predominantly MDR followed by *Klebsiella* species, *P. aeruginosa*, *S. epidermidis*, and *S. aureus.*

Recommendations

We strongly recommend to all healthcare professionals regular disinfection of inanimate objects to minimize bacterial colonization of inanimate objects and potential spread of infection, especially drug-resistant strains. Antimicrobial treatment should be based on the result of culture and sensitivity and focus should be given to the conservative and wise use of antibiotics to minimize the spreading of multi-drug resistant pathogens. Also, healthcare professionals in Debre Markos Comprehensive Specialized Hospital should focus on not carrying mobile phone with patient care materials and hospital administrators should provide infection prevention training for all healthcare professionals.

Limitations of the study

This study did not differentiate the extended beta-lactam spectrum, because of shortage of drugs. Additionally, this study excluded other important bacterial pathogens that cause HAI, such as anaerobes, due to a lack of laboratory facilities**.**

Abbreviations

AMR: Antimicrobial Resistance, APHI: Amhara Public Health Institute, ATCC: American Type Culture Collection, BSI: Bloodstream Infection, CLSI: Clinical and Laboratory Standards Institute, DMCSH: Debre Markos Comprehensive Specialized Hospital, HAI: Healthcare Associated Infection, HCP: Healthcare Professionals, ICU: Intensive Care Unit, MDR: Multidrug Resistant, MRSA: Methicillin Resistant *Staphylococcus aureus*

Acknowlegements

We would like to sincerely thank Debre Markos Comprehensive Specialized Hospital (DMCSH) administration and staffs for allowing me to conduct the research in the hospital. Our heartfelt gratitude and special thank goes to DMCSH microbiology laboratory team for providing materials and assisting the isolation and identification of the microorganisms. We acknowledge the study participants for their participation without them the research would not be a reality.

Funding

This research received no funding specific fund.

Availability of data and materials

All data information generated in this study was included in the manuscript.

Competing interests

Authors declare that they have no competing interests.

Authors' contributions

Senedu Kindie: Conceptualization, Data curation, Formal analysis, Funding acquisition, Investigation, Methodology, Project administration, Resources, Software, Validation, Visualization and Writing – original draft

Getachew Mengistu: Conceptualization, Data curation, Formal analysis, Methodology, Project administration, Supervision, Validation, Visualization and Writing – review & editing

Mulatu Kassahun: Data curation, Formal analysis, Software, Validation, and Writing– review & editing

Abebaw Admasu: Data curation, Formal analysis, Validation, Visualization and Writing– review & editing

Tebelay Dilnessa: Conceptualization, Data curation, Formal analysis, Methodology, Project administration, Supervision, Validation, Visualization and Writing– review & editing

References

1. WHO. Prevention of hospital acquired infections: A practical guid. 2002, Accessed at: http://www.who.int/csr/resources/publications/whocdscsreph 2002.pdf. Accessed on 2023/01/29.
2. WHO. The burden of health care-associated infection worldwide. 2016, Accessed at: http://www.who.int/gpsc/country_work/burden_hcai/en/ [Accessed on: 2023/01/29].
3. Akeau U. Epidemiology of Healthcare -Associated Infections, Accessed at: https://www.theific.org/wp-content/uploads/2016/04/3-Epidemiology_2016.pdf, Accessed on: 2023/01/29. 2016;Healthcare-Associated.
4. Alphons KS, Fortune TV, Haindongo E, Guillaume AYJM. Bacterial contamination and antimicrobial susceptibility from the hands of health care workers (HCWs) and inanimate surfaces in the neonatal intensive care unit (NICU) at the Windhoek Central Hospital (WCH). *Microbiol Nat*. 2020;1:83-95.
5. Curtis DE, Hlady CS, Kanade G, Pemmaraju SV, Polgreen PM, Segre AM. Healthcare worker contact networks and the prevention of hospital-acquired infections. *PLoS One*. 2013;8(12):e79906.
6. Revelas A. Healthcare–associated infections: A public health problem. *Nigerian medical journal*. 2012;53(2):59.
7. Lopez GU, Gerba CP, Tamimi AH, Kitajima M, Maxwell SL, Rose JB. Transfer efficiency of bacteria and viruses from porous and nonporous fomites to fingers under different relative humidity conditions. *Applied and environmental microbiology*. 2013;79(18):5728-34.
8. Stephens B, Azimi P, Thoemmes MS, Heidarinejad M, Allen JG, Gilbert JA. Microbial Exchange via Fomites and Implications for Human Health. *Current pollution reports*. 2019;5(4):198-213.
9. Ayalew W, Mulu W, Biadglegne F. Bacterial contamination and antibiogram of isolates from health care workers' fomites at Felege Hiwot Referral Hospital, northwest Ethiopia. *Ethiop J Health Dev*. 2019;33(2).

10. Khan M, Kashif M, Ehsan R, Faizuddin M, Iqbal S, Jamil H. Microbial contamination of white coats, hands and mobile phones of health care workers. *Intjpathol*. 2020:58-62.

11. Kotris I, Drenjanèeviæ D, Talapko J, Bukovski SJ. Identification of microorganisms on mobile phones of intensive care unit health care workers and medical students in the tertiary hospital. *Medicinski glasnik*. 2017;14(1).

12. Heyba M, Ismaiel M, Alotaibi A, Mahmoud M, Baqer H, Safar A, et al. Microbiological contamination of mobile phones of clinicians in intensive care units and neonatal care units in public hospitals in Kuwait. *BMC infect dis*. 2015;15(1):1-9.

13. Selim H, Abaza A. Microbial contamination of mobile phones in a health care setting in Alexandria, Egypt. *GMS Hyg* 2015;10.

14. Ventola C, Lee J. Mobile devices and apps for health care professionals: uses and benefits. *PT*. 2014;39(5):356.

15. Manning ML, Davis J, Sparnon E, Ballard RM. iPads, droids, and bugs: Infection prevention for mobile handheld devices at the point of care. *American journal of infection control*. 2013;41(11):1073-6.

16. Smibert OC, Aung AK, Woolnough E, Carter GP, Schultz MB, Howden BP, et al. Mobile phones and computer keyboards: unlikely reservoirs of multidrug-resistant organisms in the tertiary intensive care unit. *The Journal of hospital infection*. 2018;99(3):295-8.

17. Ustun C, Cihangiroglu M. Health care workers’ mobile phones: a potential cause of microbial cross-contamination between hospitals and community. *J Occup Environ Hyg*. 2012;9(9):538-42.

18. Burden M, Keniston A, Frank MG, Brown CA, Zoucha J, Cervantes L, *et al.* Bacterial contamination of healthcare workers' uniforms: a randomized controlled trial of antimicrobial scrubs. *J Hosp Med*. 2013;8(7):380-5.

19. Kilinc F, Selcen J. A review of isolation gowns in healthcare: fabric and gown properties. *Eng Fiber Fabr*. 2015;10(3).

20. Atay Y, Pamuk O, Boyaci B, Yildiz EZ, Göksel T, Metin DY, *et al*. A New Approach to Surgical Gowns. *Healthcare Access*. 2021.

21. Qaday J, Sariko M, Mwakyoma A, Kifaro E, Mosha D, Tarimo R, *et al.* Bacterial contamination of medical doctors and students white coats at Kilimanjaro Christian Medical Centre, Moshi, Tanzania. *IInt J Bacteriol*. 2015;2015.

22. Pilonetto M, Rosa EAR, Brofman PRS, Baggio D, Calvário F, Schelp C, *et al*. Hospital gowns as a vehicle for bacterial dissemination in an intensive care unit. *Braz J Infect Dis*. 2004;8:206-10.

23. Jain A, Shah H, Jain A, Sharma MJ. Disinfection of stethoscopes: gap between knowledge and practice in an Indian tertiary care hospital. *Ann Trop med Public Health*. 2013;6(2):236.

24. Datta P, Kaur M, Rawat S, Gupta V, Chander J. Stethoscope,"the friendly foe"–A study to evaluate bacterial contamination of stethoscopes and disinfection practices. *J Infect Dev Ctries*. 2018;12(10):887-93.

25. Saloojee H, Steenhoff A. The health professional's role in preventing nosocomial infections. *Postgrad Med J*. 2001(903):16-9.

26. Merlin MA, Wong ML, Pryor PW, Rynn K, Marques-Baptista A, Perritt R, *et al*. Prevalence of methicillin-resistant Staphylococcus aureus on the stethoscopes of emergency medical services providers. *Prehospital emergency care*. 2009;13(1):71-4.

27. Gupta A, Della-Latta P, Todd B, San Gabriel P, Haas J, Wu F, *et al*. Outbreak of extended-spectrum beta-lactamase-producing Klebsiella pneumoniae in a neonatal intensive care unit linked to artificial nails. *Infection control and hospital epidemiology*. 2004;25(3):210-5.

28. Ali S, Birhane M, Bekele S, Kibru G, Teshager L, Yilma Y, *et al*. Healthcare associated infection and its risk factors among patients admitted to a tertiary hospital in Ethiopia: longitudinal study. *Antimicrobial resistance and infection control*. 2018;7:2.

29. Messele G, Woldemedhin Y, Motuma Demissie M, Mamo K, Geyid A. Common Causes of Nosocomial Infections and Their Susceptibility Patterns in Two Hospitals in Addis Ababa. *Ethiop J Health Biomed Sci*. 2020;2(1):6.

30. Andarge A. Monitoring the urban growth of Debre Markos Town (1984-2012), Ethiopia: Using satellite images and GPS. *J Geogr Reg Plann*. 2017;10(4):69-76.

31. Ayalew W, Mulu W, F B. Bacterial contamination and antibiogram of isolates from health care workers' fomites at Felege Hiwot Referral Hospital, northwest Ethiopia. *Ethiop J Health Dev*. 2019;33(2).

32. Magiorakos AP, Srinivasan A, Carey RB, Carmeli Y, Falagas ME, Giske CG, et al. Multidrug-resistant, extensively drug-resistant and pandrug-resistant bacteria: an international expert proposal for interim standard definitions for acquired resistance. *Clinical microbiology and infection*. 2012;18(3):268-81.

33. Verma KD, Barasa A, Dara D, W/Medehen H, Asrat H, Demissie N, et al. Isolation and characterization of bacteria from mobile phones of students and employees at University of Gondar, Ethiopia. *Bull Pharm Res*. 2015;5(3):96-100.

34. Cheesbrough M. District laboratory practice in tropical countries, part 2: Cambridge University Press. 2005.

35. CLSI. Performance Standards for Antimicrobial Susceptibility Testing. 32nd ed. CLSI supplement M100. Clinical and Laboratory Standards Institute. 2022.

36. Jorgensen JH, Turnidge JD. Susceptibility test methods: dilution and disk diffusion methods. *Manual of clinical microbiology*. 2015:1253-73.

37. Segujja F, Mwambi B, Drago C, Lubowa N, Mugambwa J, Wabuyi P. Characterization and antimicrobial susceptibility patterns of isolates from ward fomites. *BBJ*. 2016.

38. Bodena D, Teklemariam Z, Balakrishnan S, Tesfa T. Bacterial contamination of mobile phones of health professionals in Eastern Ethiopia: antimicrobial susceptibility and associated factors. *Tropical medicine and health*. 2019;47:15.

39. Akanbi AAA, Kareem T, Adedoja A, Nyamngee A, Muhammed MBU, Abdulkareem K, et al. Bacterial contamination of medical doctors' white coats as contributing factor to hospital acquired infections. *Int J Biol Chem Sci*. 2017;11(1):185-94.

40. Maryam A, Hadiza U, Aminu U. Characterization and determination of antibiotic susceptibility pattern of bacteria isolated from some fomites in a teaching hospital in northern Nigeria. *Afr J Microbiol Res*. 2014;8(8):814-8.

41. Rao DA, Aman A, Muhammad Mubeen S, Shah A. Bacterial contamination and stethoscope disinfection practices: a cross-sectional survey of healthcare workers in Karachi, Pakistan. *Tropical doctor*. 2017;47(3):226-30.

42. Maqsud A, Alsuhaymi A, Fallatah K, Al-johany K, Sabr M, Alharbi W, *et al*. The Prevalence of Stethoscopes Contamination at Tertiary Center in AL Madinah Almonawwarah Saudi Arabia 2015. *IJASR*. 20115;4(1):103-10.

43. Haque MA, Alam KMF, Sharmin LS, Mahmud A, Parvez MW, Rahman MK, et al. Bacterial Contamination of Stethoscope in a Tertiary Care Hospital. *TAJ*. 2017;30(2):1-6.

44. Chaka T, Misgana G, Feye B, Kassa R. Bacterial isolates from cell phones and hands of health care workers: a cross sectional study in pediatric wards at Black Lion Hospital, Addis Ababa, Ethiopia. *J Bacteriol Parasitol*. 2016;7(288):2.

45. Misgana G, Abdissa K, Abebe G. Bacterial contamination of mobile phones of health care workers at Jimma University Specialized Hospital, Jimma, South West Ethiopia. *Int J Infect Contro*. 2015;11(1).

46. Gashaw M, Abtew D, Addis Z. Prevalence and Antimicrobial Susceptibility Pattern of Bacteria Isolated from Mobile Phones of Health Care Professionals Working in Gondar Town Health Centers. *International Scholarly Research Notices*. 2014;2014(1):205074.

47. Pareek V, Mishra S, Chauhan D, Tripathi S. Mobile phones of healthcare workers: A potent reservoir of bacterial nosocomial infection. . *IJMER*. 2021.

48. Galazzi A, Panigada M, Broggi E, Grancini A, Adamini I, Binda F, et al. Microbiological colonization of healthcare workers' mobile phones in a tertiary-level Italian intensive care unit. *Intensive Crit Care Nurs*. 2019;52:17-21.

49. Yao N, Yang X-F, Zhu B, Liao C-Y, He Y-M, Du J, *et al.* Bacterial Colonization on Healthcare Workers' Mobile Phones and Hands in Municipal Hospitals of Chongqing, China: Cross-contamination and Associated Factors. *J Epidemiol Glob Health*. 2022;12(4):390-9.

50. Edrees H, Al-Awar S. Bacterial contamination of mobile phones of medical laboratory workers at Sana'a city, Yemen and their antimicrobial susceptibility. *JPPRes*. 2020;8(6):591-9.

51. Al-Beeshi N, Alohali R, Torchyan A, Somily A. The bacterial colonization of healthcare workers' mobile phones in a large tertiary care teaching hospital in Saudi Arabia. *J Infect Dev Ctries*. 2021;15(09):1314-20.

52. Bakry M, Attia T, Amer O, Asaad AM. Bacterial Contamination of Healthcare Workers' Mobile Phones in Pediatric and Neonatal Departments of Two Hospitals in Zagazig City. *ZUMJ*. 2020;26(3):384-96.

53. Mishra SK, Maharjan S, Yadav SK, Sah NP, Sharma S, Parajuli K, et al. Bacteria on Medical Professionals' White Coats in a University Hospital. *The Canadian journal of infectious diseases & medical microbiology*. 2020;2020:5957284.

54. Haun N, Hooper-Lane C, Safdar N. Healthcare personnel attire and devices as fomites: a systematic review. *Infect Control Hosp Epidemiol*. 2016;37(11):1367-73.

55. Dabsu R, Woldeamanuel Y, Asrat D. Otoscope and stethoscope: Vehicles for microbial colonization at Tikur Anbessa Specialized Referral Hospital, Addis Ababa, Ethiopia. *Ethiop J Health Dev*. 2014;28(1).

56. Salile T. Bacteriological assessment of stethoscope used by health care personnel in attat hospital, SNNP, Gurage Zone, Ethiopia. *IJAMB*. 2021;4(2).

57. Lokkur P, Nagaraj SJ. The prevalence of bacterial contamination of stethoscope diaphragms: a cross sectional study, among health care workers of a tertiary care hospital. *Indian J Med Microbiol*. 2014;32(2):201.

58. Shiferaw T, Beyene G, Kassa T, Sewunet T. Bacterial contamination, bacterial profile and antimicrobial susceptibility pattern of isolates from stethoscopes at Jimma University Specialized Hospital. *Annals of clinical microbiology and antimicrobials*. 2013;12:39.

59. Girma G. Potential health risks with microbial contamination of mobile phones. *GRJE*. 2015;3(1):246-54.

60. Asfaw T, Genetu DJ. High Rate of Bacterial Contamination on Healthcare Worker’s Mobile Phone and Potential Role in Dissemination of Healthcare-Associated Infection at Debre Berhan Referral Hospital, North Shoa Zone, Ethiopia. *Risk Manag Healthc Policy*. 2021;14:2601.

61. Pal K, Chatterjee R, Biswas A, Samanta AK. Bacterial contamination and disinfection of stethoscopes: a knowledge gap among health care personnel of a tertiary care hospital of rural Bengal. *IOSR-JDMS*. 2015;14(7):44-9.

62. Worku T, Derseh D, Kumalo A. Bacterial Profile and Antimicrobial Susceptibility Pattern of the Isolates from Stethoscope, Thermometer, and Inanimate Surfaces of Mizan-Tepi University Teaching Hospital, Southwest Ethiopia. *International journal of microbiology*. 2018;2018:9824251.

63. Morubagal RR, Shivappa SG, Mahale RP, Neelambike SM. Study of bacterial flora associated with mobile phones of healthcare workers and non-healthcare workers. *Iranian journal of microbiology*. 2017;9(3):143-51.

64. Sahiledengle B. Stethoscope disinfection is rarely done in Ethiopia: what are the associated factors? *PloS one*. 2019;14(6):e0208365.

65. Weldegebreal F, Admassu D, Meaza D, Asfaw M. Non-critical healthcare tools as a potential source of healthcare-acquired bacterial infections in eastern Ethiopia: a hospital-based cross-sectional study. *SAGE* 2019;7:2050312118822627.

66. Thapa S, Sapkota LB. Bacteriological assessment of stethoscopes used by healthcare workers in a tertiary care centre of Nepal. *BMC research notes*. 2017;10(1):353.

67. Tiwari A, Ankola AV, Mishra H, Kakkar M. Assessment of bacterial contamination in cellular phones of dental professionals working in a dental institution in Belgaum city–a cross sectional study. *Med Res Chron*. 2016;3(03):266-73.

68. Darge A, Kahsay AG, Hailekiros H, Niguse S, Abdulkader M. Bacterial contamination and antimicrobial susceptibility patterns of intensive care units medical equipment and inanimate surfaces at Ayder Comprehensive Specialized Hospital, Mekelle, Northern Ethiopia. *BMC research notes*. 2019;12(1):621.

69. Firesbhat A, Tigabu A, Tegene B, Gelaw B. Bacterial profile of high-touch surfaces, leftover drugs and antiseptics together with their antimicrobial susceptibility patterns at University of Gondar Comprehensive Specialized Hospital, Northwest Ethiopia. *BMC* 2021;21(1):1-13.

70. Mushabati N, Samutela M, Yamba K, Ngulube J, Nakazwe R, Nkhoma P, *et al*. Bacterial contamination of mobile phones of healthcare workers at the University Teaching Hospital, Lusaka, Zambia. *Infection prevention in practice*. 2021;3(2):100126.

71. Bayraktar M, Kaya E, Ozturk A, İbahim BMS. Antimicrobial susceptibility of bacterial pathogens isolated from healthcare workers' cellphones. *Infect Dis Now*. 2021;51(7):596-602.

72. Nivedhitha E, Duraivel M, Kayalvili K, Selvan SA. Study to Assess the Risk of Transmission of Microbial Organisms and their Resistance Pattern on Dresses and Stethoscopes of Health Care Workers. *J Pure Appl Microbiol*. 2021;15(3):1150-6.

73. Aftab H, Zia B, Zahid M, Raheem A, Beg M. Knowledge, Attitude, and Practices of Healthcare Personnel Regarding the Transmission of Pathogens via Fomites at a Tertiary Care Hospital in Karachi, Pakistan. *OFID*. 2016;3(1):208.

74. Birru M, Mengistu M, Siraj M, Aklilu A, Boru K, Woldemariam M, *et al.* Magnitude, Diversity, and Antibiograms of Bacteria Isolated from Patient-Care Equipment and Inanimate Objects of Selected Wards in Arba Minch General Hospital, Southern Ethiopia. *Research and reports in tropical medicine*. 2021;12:39-49.

75. Kumar PA, Chougale RA, Sinduri I. Bacterial Contamination of White Coats among Medical Personnel-A Cross Sectional Study in Kolhapur, India. *J Pure Appl Microbiol*. 2020;14(2):1405-11.

76. Shahlol A, Khalifallah H, Shahlol E, Dastidar R, Halder M, Lamichhane G. Bacterial contamination of mobile phones and hands of health care workers in Sabha Medical Center Hospital, Fazzan area in southwestern of LIBYA. *Int J Curr Res Med Sci* 2015;1(4):1-8.

77. Bitew K, Gidebo DD, Ali MM. Bacterial contamination rates and drug susceptibility patterns of bacteria recovered from medical equipment, inanimate surfaces, and indoor air of a neonatal intensive care unit and pediatric ward at Hawassa University Comprehensive Specialized Hospital, Ethiopia. *IJID regions*. 2021;1:27-33.

78. Ogheneochuko A. Microbial Colonization and Multi Drug Resistance Patterns of Phones of Health Care Workers: Potential for Nosocomial Pathogens Transmission in Delta State, Nigeria. *IJ MIC and Biot*. 2022.

79. Pal K, Chatterjee R, Biswas A, Samanta AK. Bacterial contamination and disinfection of stethoscopes: a knowledge gap among health care personnel of a tertiary care hospital of rural Bengal. *IOSR-JDMS*. 2015;14(7):44-9.

Figure 1

Click here to access/download;Figure;Fig 1.tif

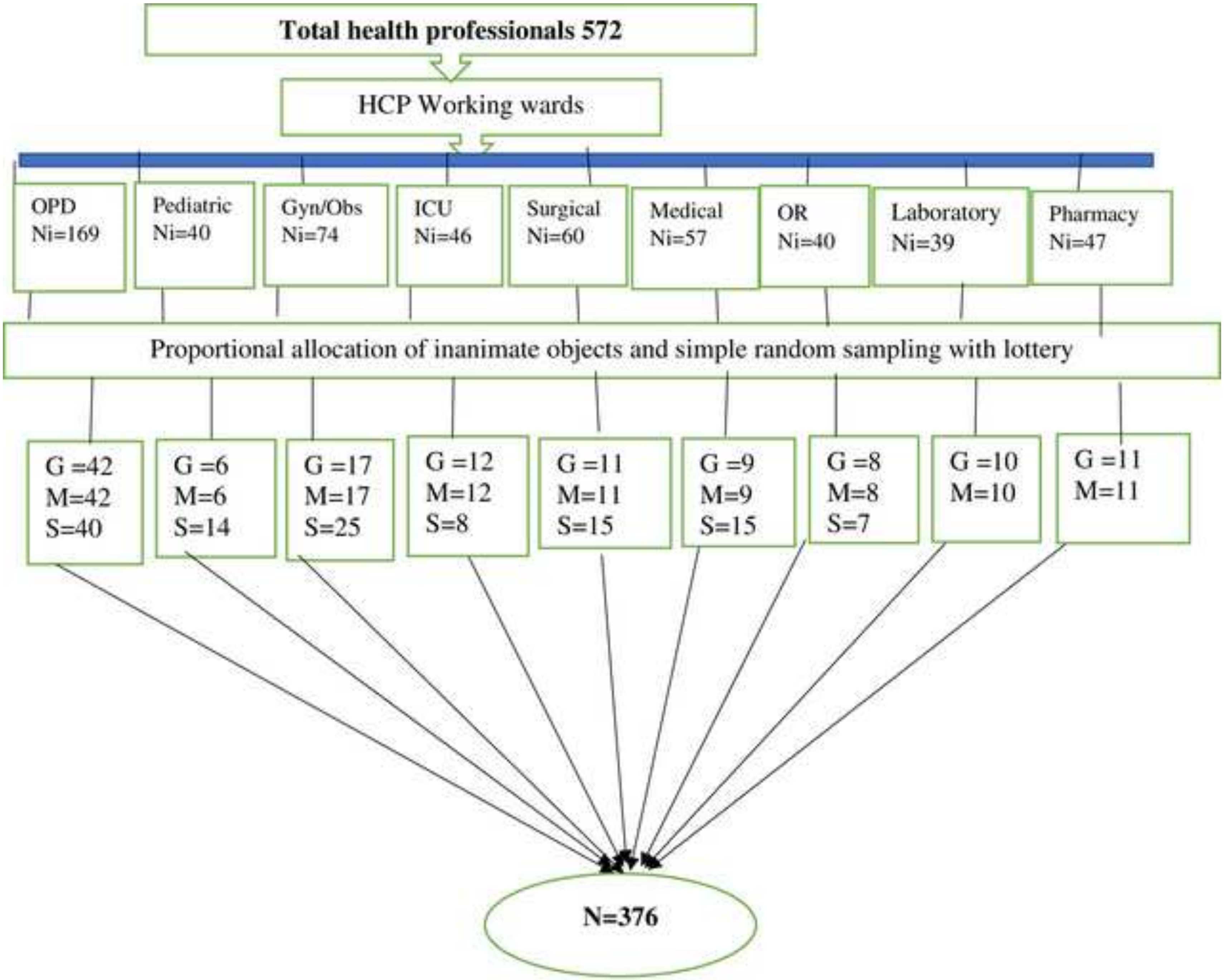

Key: - G=gown, M=mobile phone, S=stethoscope

Figure 2

Click here to access/download;Figure;Fig 2.tif

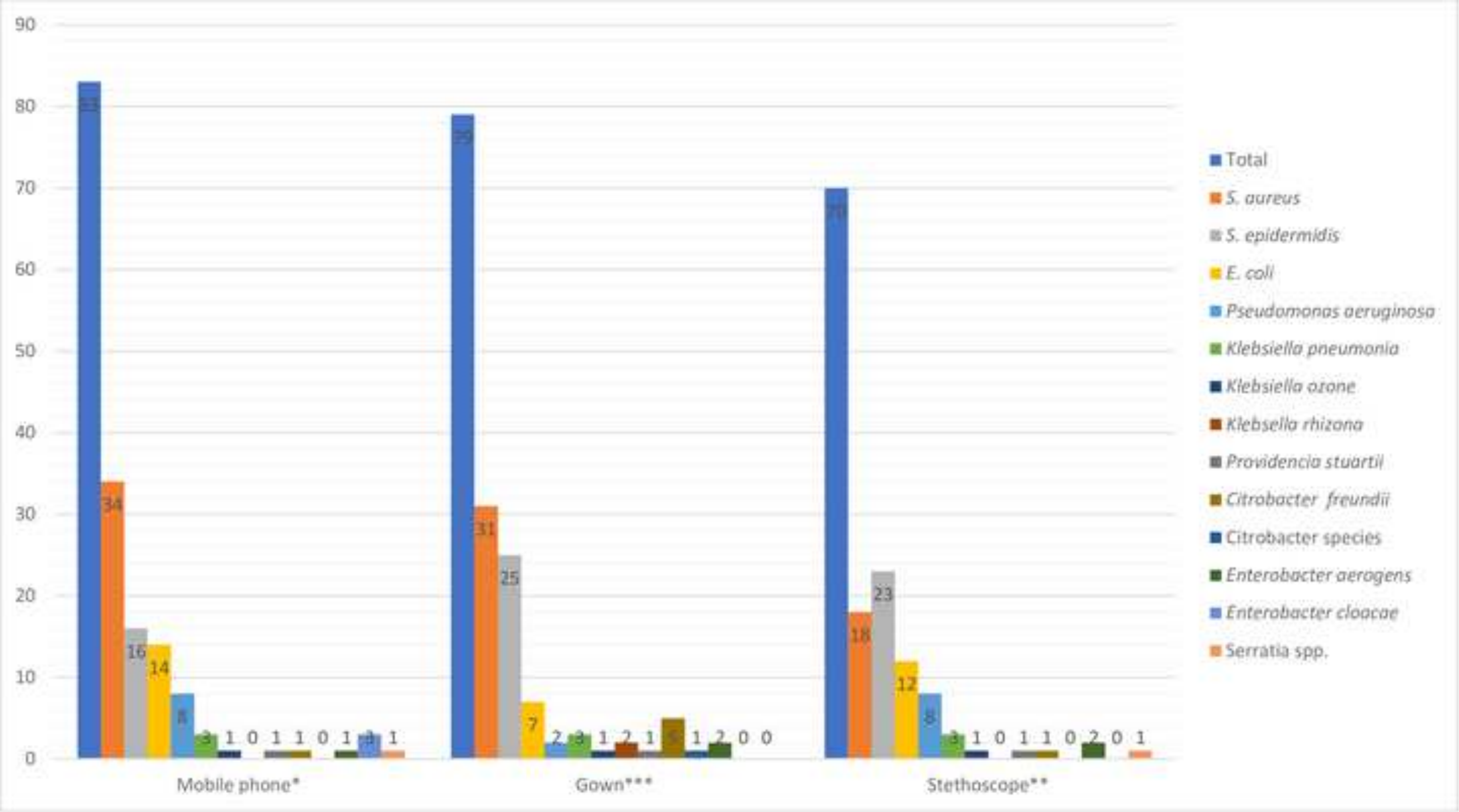

***: Three gowns co-infected with two bacteria, **: One stethoscope co-infected with two bacteria, *: One mobile phone co-infected with two bacteria

Click here to access/download
Supporting Information
Suplementary file 1.docx

Click here to access/download
Supporting Information
Suplementary file 2.docx

Ethical clearance

Click here to access/download
Supporting Information
Ethical Clearance.pdf
